# A Model of the Full-Length Cytokinin Receptor: New Insights and Prospects

**DOI:** 10.3390/ijms25010073

**Published:** 2023-12-20

**Authors:** Dmitry V. Arkhipov, Sergey N. Lomin, Georgy A. Romanov

**Affiliations:** Timiryazev Institute of Plant Physiology, Russian Academy of Sciences, Botanicheskaya 35, 127276 Moscow, Russia; hotdogue@yandex.ru (D.V.A.); losn1@yandex.ru (S.N.L.)

**Keywords:** cytokinin signaling, cytokinin receptor structure, histidine kinase, phytohormones, molecular modeling, AlphaFold Multimer, ColabFold, Arabidopsis, potato

## Abstract

Cytokinins (CK) are one of the most important classes of phytohormones that regulate a wide range of processes in plants. A CK receptor, a sensor hybrid histidine kinase, was discovered more than 20 years ago, but the structural basis for its signaling is still a challenge for plant biologists. To date, only two fragments of the CK receptor structure, the sensory module and the receiver domain, were experimentally resolved. Some other regions were built up by molecular modeling based on structures of proteins homologous to CK receptors. However, in the long term, these data have proven insufficient for solving the structure of the full-sized CK receptor. The functional unit of CK receptor is the receptor dimer. In this article, a molecular structure of the dimeric form of the full-length CK receptor based on AlphaFold Multimer and ColabFold modeling is presented for the first time. Structural changes of the receptor upon interacting with phosphotransfer protein are visualized. According to mathematical simulation and available data, both types of dimeric receptor complexes with hormones, either half- or fully liganded, appear to be active in triggering signals. In addition, the prospects of using this and similar models to address remaining fundamental problems of CK signaling were outlined.

## 1. Introduction

**Figure 1:**
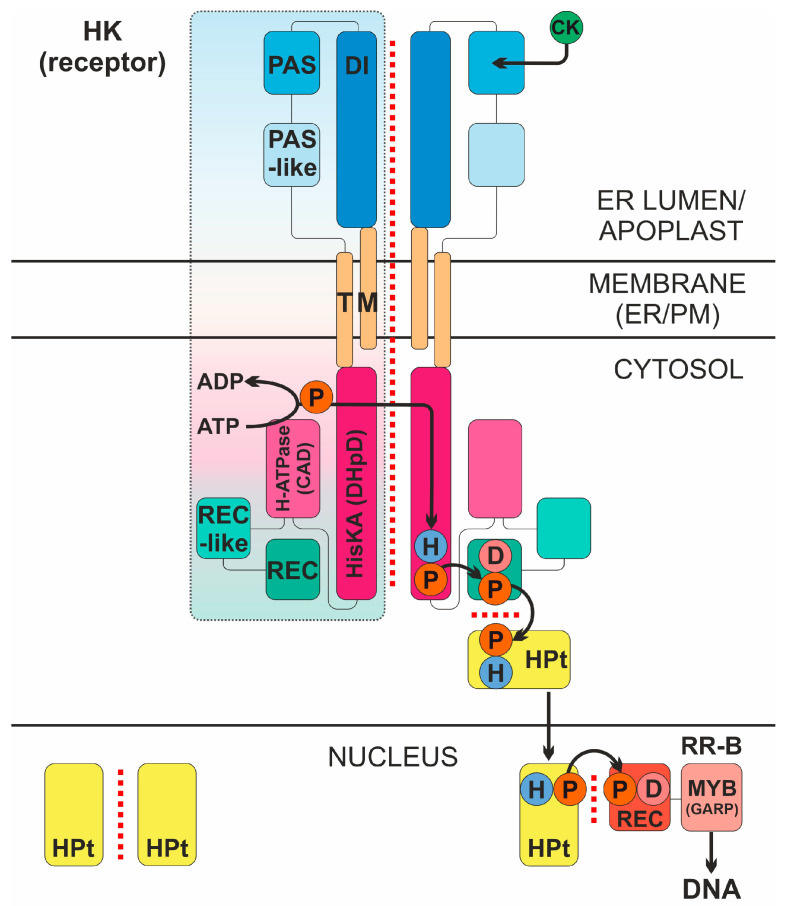
Intermolecular interactions in the cytokinin signal transduction system. CK, cytokinin; HK, CK receptor; P, phosphate; D, conserved aspartate; H, conserved histidine; DI, dimerization interface region of the sensory module (SM); PAS and PAS-like are subdomains of CHASE domain of the SM; TM, transmembrane domains; HisKA (DHpD), histidine kinase domain; H-ATPase (CAD), adenosine triphosphatase domain; REC-like, pseudo-receiver domain; REC, receiver domain; HPt, phosphotransmitter; RR-B, type B response regulator (transcription factor); ER/PM, endoplasmic reticulum/plasma membrane. Protein–protein interactions are indicated by red dotted line. The signaling mechanism is realized as follows. High-affinity hormone binding to SM triggers autophosphorylation in *trans* of conserved His, followed by “hot” phosphoryl group transfer in *cis* to conserved Asp in the REC domain at the C-terminus of the receptor. Next, the “hot” phosphate is transmitted to conserved His of the HPt protein, which cycles between cytoplasm and nucleus providing phosphate to conserved Asp of the nuclear RR-B protein. After acquiring phosphoryl group, RR-B is activated and changes (usually upregulates) the expression of primary response genes.

Cytokinins (CKs) are classical phytohormones with a wide spectrum of physiological action. They are involved in processes such as the stimulation of cell division, the maintenance of the shoot apical meristem, xylem development, the inhibition of root growth, senescence retardation, root-to-shoot signaling, nutrient assimilation, tuberization, and nodulation in legumes [1,2]. In addition, CKs are involved in protecting plants from biotic and abiotic stresses and perform other functions in the plant organism [3,4,5]. CK signaling occurs via the histidine-aspartate pathway (Figure 1), using the so-called multistep phosphorelay (MSP), a modified prokaryotic two-component system (TCS). The CK signaling machinery consists of three main types of proteins: the CK receptor—the sensor histidine kinase (HK); the phosphotransmitter (HPt); and the type B response regulator (RR-B). Each of the stages of signal transmission involves protein–protein interactions [6,7]: the dimerization of receptors, histidine phosphorylation in *trans*, the interaction of receptors with HPts, the dimerization of HPts themselves, and their interaction with response regulators.

The CK receptor, a key player In CK signaling, is a complex multidomain transmembrane protein. In *Arabidopsis thaliana*, three CK receptors were identified: AHK2, AHK3, and AHK4 (CRE1) [8,9,10]. Their ligand-recognizing sensory module (SM) localizes on the extracytosolic side of the membrane [11] and consists of a subdomain forming the dimerization interface (DI) and a CHASE (Cyclases/Histidine kinases Associated SEnsory) domain [12,13,14], including Per-Arnt-Sim (PAS) and pseudo-PAS (or PAS-like) subdomains [14,15]. SM is flanked at both sides by transmembrane (TM) domains. It can occur in up to four TM domains upstream (located before the N-terminus of SM), but only one such domain downstream (located after the C-terminus of SM) of CHASE. The total number of TM domains is usually maximal in AHK2 and minimal in AHK4 receptor subfamilies [14]. The cytosolic part of the receptor, located on the opposite side of the membrane, includes a catalytic module consisting of the HisKA (also called DHpD—Dimerization and Histidine phosphotransfer Domain) and H-ATPase (also called CAD—Catalytic and ATP-binding Domain) domains linked to a receiver module combining the receiver (REC, or RD) and pseudo-receiver (REC-like, or RLD) domains [10,16].

Modeling the complete dimeric structure of CK receptors is important for understanding the key functional aspects of CK signaling. To date, only two fragments of receptor structure have been experimentally resolved: *A. thaliana* AHK4 SM in complexes with seven cognate ligands [15] and, more recently, AHK4 and *Medicago truncatula* MtCRE1 RDs (REC) [17]. In addition to CK receptors, histidine kinases are represented in Arabidopsis by some ethylene receptors (ETR1 and ERS1), as well as AHK1, CKI1, and AHK5 (CKI2) proteins [18,19]. There are also a plethora of sensor histidine kinases in bacteria, archaea, and fungi homologous to CK receptors [20,21]. Notably, even if they resemble a 3D structure, they can still differ significantly in the primary structure [15]. Disclosure of the spatial structures of such homologs makes it possible to extrapolate to CK receptors the mechanism of their functioning and observed functional states.

It is possible to envisage many conformations corresponding to various functional states of CK receptors. The basic state is the unliganded one, namely, the apo-form lacking “hot” phosphoryl on conserved amino acids (aa). This conformation is certainly non-active and could serve as an initial structure for comparison with receptors in the active state. Unfortunately, this apo-form has not yet been solved to date. The other states correspond to liganded CK-bound conformations: the holo-form, in the unphosphorylated state, with ATP in the substrate-binding site of the H-ATPase domain; the conformation with phosphorylated conserved histidine in the catalytic module; that with phosphoaspartate in RD after phosphotransfer; and that with phosphorylated RD ready to transfer phosphate to HPt. It should be noted that the variety of potential conformations is actually very large, since the conformational status of subunits in the given receptor dimer can be different and unrelated to each other, so a lot of combinations may be expected.

Recent advances in molecular modeling technology, in particular the emergence of programs based on artificial intelligence, made it possible to solve the 3D structure of various proteins, regardless of their size and availability of templates close in the primary structure [22]. Along with traditional methods like homology modeling, molecular docking, and molecular dynamics, these programs provide impressive insights into the structure and dynamics of large complex proteins. The use of artificial intelligence-based software, in particular AlphaFold [22] or ColabFold [23], allowed us to build a scientifically substantiated 3D model of the full-length CK receptor dimer (see Section 3) and its complex with HPt protein. As far as we know, a model of the full-length CK receptor in dimeric form, as well as a model of its complex with HPt, are demonstrated here for the first time.

## 2. Background: Structural and Functional Features of CK Signaling Components and Their Homologs

### 2.1. Structure and Functions of Sensory Modules and PAS Domains 

The spatial organization of AHK4 SM is well known due to the solving of its crystal structure [15]. As already mentioned, the SM of CK receptors consists of the DI and CHASE domain, which includes PAS and PAS-like (sub)domains (Figure S1) [14]. The average sizes of PAS and PAS-like domains correspond to 100 and 70 aa residues, respectively. The canonical structure of PAS domains represents a β-sheet consisting of five antiparallel β-strands and four α-helices [24,25]. The PAS domain consists of several functional parts: N-terminal cap, PAS core, helical connector, and β-scaffold (Figure S2a) [26,27,28]. The spatial structure of CK receptors SM resembles many bacterial sensory domains, despite the low percentage of aa sequence identity (Figure S3) [20]. However, the SM of CK receptors also has some peculiarities, particularly its membrane-proximal PAS domain, which is degenerated in structure and is therefore considered to be PAS-like. The ligand-binding membrane-distal PAS domain, however, possesses an additional structural element relative to the canonical PAS, namely the “upper” region of PAS (Figures S1a, S2b and S3) [14]. Moreover, AHK2 and AHK3 receptor orthologs have bulkier “upper” region relative to AHK4 orthologs. For example, in potato (*Solanum tuberosum*), the receptors StHK2 and StHK3 differ from StHK4 by insertions of 14 and 17 aa residues, respectively [16].

Functionally, about 20 aa residues of the PAS domain make up the ligand binding site of the CK receptor [15,29]. These residues form specific hydrogen bonds and hydrophobic interactions that provide high affinity CK binding. Although CK receptors differ significantly from each other in ligand preference, most of them bind *trans*-zeatin, the main cytokinin, with a dissociation constant of 1–10 nM. These values correspond well to the experimentally detected cytokinin concentrations in planta [29,30].

The crystal structure of AHK4 has only been obtained in the ligand-bound form, so the apo-form of the receptor SM is still missing, impairing the disclosure of the dynamic changes in the receptor molecule that are induced by hormone binding [15]. However, many of the structural homologs of SM crystallized in both ligand-bound and apo-forms, demonstrating several types of conformational changes in the sensory domains caused by cognate ligand binding (Figure S4) [31,32]. A linear displacement of one dimer subunit or its part relative to another one is an example of one such movement. This movement along the membrane normal vector was dubbed piston-type. The piston type is typical for the Bistris-complexed mmHK1s-Z2 of the anaerobic archaea *Methanosarcina mazei* protein [31] and chemoreceptor Tar (*Salmonella typhimurium*) [33]. Another type of movement is the rotational one, which includes two variants. In one case, known as “domain rotation”, one half of the sensory domain deviates from the common central axis after the ligand binding by the distal PAS domain. This is a feature of the *Vibrio parahaemolyticus* vpHK1S-Z8 protein, for example [31]. An important detail is the closing of the ligand-binding pocket by the loop after ligand binding in vpHK1S-Z8, but not mmHK1S-Z2 (*Methanosarcina mazei*). In another variant of the rotational type, called “helical rotation”, the α-helix is rotated around its axis after ligand binding to the SM. An instance of such a variant is the *Vibrio harveyi* LuxP–LuxQ complex [34]. The third type of the movement is scissor-like, which implies a simultaneous diagonal deviation of the subunits (the edges of involved helical segments moving in opposite directions) upon ligand binding to the SM [32]. This variant has been shown for the sensory domains of the heparin binding hybrid kinase BT4663 (*Bacteroides thetaiotaomicron*) [35] and the magnesium-sensitive PhoQ (*Escherichia coli*) [36]. It should also be noted the change in the area of the dimerization interface between apo- and holo-forms. In the structure of the liganded DctB-dimer (*Rhizobium meliloti*), the N-terminal helices α2 and α3 and the upper half of α1 make up most of the dimerization interface. In the DctB apo-form, the α5-helix and the N-terminal half of the α1 helix also participate in dimerization. As a result, the dimerization surface area in the apo-form encompasses 1130 Å^2^, whereas the corresponding area in the dimer of DctB-succinate complex is reduced by about half to 540 Å^2^. Thus, when succinate binds to the DctB dimer, α1-helices diverge in the membrane-proximal portion in a scissor-like fashion. It was shown that succinate binding also causes conformational changes in the β3–β4 loop of the membrane-distal PAS domain [37]. In ligand-bound CK receptors, according to the crystal structure of AHK4, the SM dimerization interface is also formed only in the membrane-distal part [15]; this makes AHK4 and DctB dimeric proteins related. The dimerization properties of SM (such as calculated interaction energies, aa residues determining dimerization, conservation, hydrophobicity, electrostatic potential, and complementarity in dimerization interfaces) in this conformation have been described in detail [7].

### 2.2. Structure and Functions of Catalytic Modules (HisKA and H-ATPase Domains) 

For the next functional part of the CK receptor—the catalytic module—experimentally solved structures are not available. Crystal structures of its homologs, the ethylene receptors of *A. thaliana*—namely the HisKA domain of ERS1 and the ATP-binding domain of ETR1—have been published [38]. In addition, the PDB database contains several structures of cytosolic parts of bacterial and fungal histidine kinases that are homologous to CK receptors varying degrees. Among the published structures crystallized at different stages of the catalytic cycle are bacterial H-ATPase domains (CAD) containing either an ATP molecule in the active center, such as ShkA from *Caulobacter vibrioides* (PDB ID: 6QRJ) [39], or an ADP molecule, such as the HK853-RR468 complex from *Thermotoga maritima* (PDB ID: 4JAS) [40].

The HisKA domain of the ERS1 receptor was crystallized as a dimer (PDB IDs: 4MT8, 4MTX) with a hairpin-shaped monomer formed by two antiparallel α-helices connected by a short loop [38]. The N-terminal α2-helix is about 95 Å long, which is significantly longer than most known bacterial DHpD structures. The C-terminal α-helix of the ERS1 is considerably shorter, being about 40 Å long. At the site corresponding to the first 30 aa residues, the α1-helices of the two dimer subunits form a parallel left-handed helical coil. The rest of the “hairpin” subunits of the dimeric HisKA domain form a four-helix bundle.

The structure of the catalytic ATP-binding domain (H-ATPase or CAD) is an α/β-sandwich, one layer of which is formed by a mixed (parallel/antiparallel) β-sheet consisting of five β-strands, whereas the second layer includes three α-helices and a pair of short antiparallel β-strands. The ADP/ATP molecule is located in the nucleotide-binding site of the CAD and interacts with the conserved aa residues of the N, G1, F, G2, and G3 boxes, the purine ring of ADP/ATP facing a hydrophobic pocket (Figure S5) [38,41,42]. The crystal structure of the catalytic ATP-binding domain of ETR1 corresponds to the classical structure of H-ATPase domains, adjusting for the fact that two loops are missing, including the “ATP cap” that closes the entrance to the nucleotide-binding pocket [38].

A large insert (more than 50 aa residues long) in the β2–β3 linker of the H-ATPase domain distinguishes CK receptors from bacterial histidine kinases and the H-ATPase domain of the ethylene receptor [16]. This insertion is located on the side opposite to the ATP binding site. To establish the possible structure of the insert in the β2–β3 linker, de novo modeling of the H-ATPase structure of the StHK3 receptor domain has been performed using the IntFOLD service [43] and compared with the homology model. In one of the best versions of the resulting model, the insertion includes three α-helices [16], which partially correlates with the structure of the H-ATPase domain of histidine kinase CckA (*Caulobacter crescentus*), where shorter insert forms an α-helix too [44].

Histidine kinases can function by *cis*- or *trans*-autophosphorylation (Figure S6). It is assumed that CK receptors are *trans*-phosphorylated [29,45]. In the case of *cis*-phosphorylation (intrasubunit), the CAD of subunit A transfers phosphate to the DHpD of subunit A; in the case of *trans*-phosphorylation (inter-subunit), phosphate transfer occurs between subunits A and B. It was shown that the ability of the histidine kinase to undertake one or another form of autophosphorylation is determined by the loop in the proximal DHpD connecting the α1 and α2 helices [46]. A model has been proposed in which the orientation of the loop connecting the helices determines whether phosphorylation will take place in the *cis*- or *trans*-type. The EnvZ of *E. coli* has a right-handed loop and is autophosphorylated in the *trans*-type, while the HK853 of *T. maritima* has a left-handed loop and is autophosphorylated in the *cis*-type [46].

The determination of several sets of crystal structures of histidine kinase DesK from *Bacillus subtilis* and the DesK–DesR complex in various states made it possible to describe the mechanism of switching histidine kinase from phosphatase to phosphotransferase activity [47,48,49]. The switching mechanism is based on the possibility of rotation of the α-helices of the DHpD’s dimer four-helix bundle around their axes (Figure S7). In the phosphatase state, the α-helices of the DHpD domain of DesK are oriented in such a way that the side chain of the conserved phosphoaccepting histidine (His188) is directed inside the helix bundle and is inaccessible for phosphorylation. In the phosphotransferase state, the α-helices of DHpD DesK are rotated so that His188 faces outward and is able to accept phosphate. At the same time, before the start of the reaction, His188 is bound to Asp189 of the same subunit by hydrogen bonding. Once the CAD approaches the DHpD and phosphate is transferred from ATP to His188, the histidine rotamerizes, causing the Asp189 binding to be lost and creating a conformation suitable for phosphate transfer to the RD of the response regulator [48]. The AHK4 receptor is known to exhibit phosphatase activity in the absence of CK, and it is likely that this switching mechanism can also function here [29,50].

### 2.3. Structure and Functions of Receiver Modules (Receiver and Pseudoreceiver Domains) 

The third functional unit of the CK receptor is the receiver module. In addition to the RD itself, CK receptors also have pseudoreceiver domain, that resembles RD in its spatial structure [16]. A large number of RD structures have been published, including the CK receptors and their homologs from plants, bacteria and fungi. The structures of the following Arabidopsis RDs have been resolved: CK receptor AHK4 (CRE1) (PDB IDs: 7P8C, 7P8D) [17], ethylene receptor ETR1 (PDB ID: 1DCF) [51], other histidine kinases (non-receptors) CKI1 (PDB ID: 3MMN) [52], and CKI2(AHK5) (PDB ID: 4EUK) [53].

RDs have a (β/α)_5_ fold type and are formed by a β-sheet consisting of five parallel β-strands, which is framed by five α-helices: two on one side of the sheet and three on the other side (Figure S8a). There are RDs that include additional structure elements beyond the standard (β/α)_5_ fold. Thus, AHK5_RD_ (synonymous with CKI2_RD_) (PDB ID: 4EUK) [53] contains six α-helices instead of the five usually observed in RDs. An additional α4-helix is located on the side opposite to the active site and the interface of interaction with the HPt. The region including this helix contains about 25 additional aa relative to ETR1_RD_, CKI1_RD_ and SLN1_RD_ (Figure S8b). According to molecular modeling, such a small additional helix is present in the RD of three potato (StHK2-4) and two Arabidopsis (AHK2,3) receptors, while in AHK4_RD_, the fold is standard, with only five α-helices [7,16].

The conserved aa residues of the active site of the RD are located at the C-terminus of the three central β-strands of the β-sheet (β1, β3 and β4). The β1-chain is followed by two conserved aspartate residues (the first may in some cases be replaced by glutamate), which take part in the binding Mg^2+^ ion. A highly conserved phosphoaccepting aspartate is located at the C-terminus of the β3-chain [54]. In addition to the active center itself, several more regions of the RD play an important role in its functioning. One of these sites is a highly conserved lysine located at the C-terminus of the β5 chain, which contributes to conformational changes during phosphorylation [52,54]. Another important site is the β3–α3 loop (also called the γ-loop), which forms a 180° γ-turn provided by a highly conserved proline [51,54].

The crucial aspect of RD functioning is its interaction with HPt. *A. thaliana* AHK5_RD_ was crystallized in a complex with AHP1 and Mg^2+^ ion in the active site (PDB ID: 4EUK) [53]. AHP1 has a structure typical of plant HPt’s and consists of six α-helices. Four C-terminal helices form an antiparallel four-helix bundle, with two of these helices (α3 and α6) being relatively long while the remaining two (α4 and α5) are shorter.

The AHK5_RD_–AHP1 complex demonstrated some peculiarities of the interaction of plant HPts with RD. The RD α1-helix fits into a recess defined by three α-helices (α2–4) and the adjacent L2 loop in HPt, forming a rectangular contact pad. One half of the interface includes predominantly hydrophobic contacts, and the other half is formed by polar residues, which in AHP1 are arranged in a ridge and participate in hydrogen bonding with aa of AHK5_RD_. The conserved phosphoacceptor histidine (His79 in AHP1) is located at the very edge of the interface and is mostly solvent accessible [53]. More recently, complexes of Arabidopsis and potato CK receptors RDs with HPt’s were constructed by homology modeling and studied in detail [7]. It should also be noted the importance of phosphorylation and magnesium ion in the active center of the RD on its interaction with HPt [7,17,52,55].

For a long time, the structure and function of pseudoreceiver domains, which are present in some hybrid histidine kinases (including CK receptors) and absent in others (e.g., ethylene receptors), remained unknown. The primary structure of the pseudoreceiver domains is the most variable relative to other receptor domains; however, it retains a number of sites characteristic of the REC superfamily. According to molecular modeling, the pseudoreceiver domains of potato receptors have a folding typical of the REC superfamily, which includes five parallel β-strands and five α-helices [16].

The publication of the structure of the full-length *Caulobacter vibrioides* ShkA protein revealed more about the structure and function of pseudoreceiver domains (Figure S9) [39]. This protein is a soluble hybrid histidine kinase and includes four domains—DHpD, CAD, Rec1 (pseudo-receiver), and Rec2 (receiver)—which corresponds to the domain organization of the cytosolic part of cytokinin receptors. The Rec1 pseudo-receiver domain (which cannot participate in phosphotransfer) is tightly associated with the catalytic CAD, while to the Rec2 Rec1 is connected through a long partly disordered linker. Rec2 interacts with Rec1 with the participation of the DDR motif, which is located on the Rec1–Rec2 linker, in close proximity to Rec2. Histidine kinase in this conformation is autoinhibited and inactive, since Rec2 prevents the convergence of the CAD and DHpD and, consequently, the transfer of phosphate from the catalytic center to the conserved histidine. When c-di-GMP is present, it competes with the DDR motif for the Rec1 binding site and, thus, breaks the Rec1–Rec2 interaction, after which Rec2, due to the long Rec1–Rec2 linker, frees up the space between the CAD and DHpD, providing possibility of autophosphorylation [39].

## 3. The Model of Full-Length Cytokinin Receptors

### 3.1. Previous Advances in Modeling a Full-Length CK Receptor

Solving the complete structure of the CK receptor has always been an important task for researchers, which has remained unrealized for a long time. As stated in the Introduction, for many years, the only experimentally solved structure was the AHK4 sensory module (SM) [15] in complexes with various CKs. Structures of the dimeric form of the isolated *A. thaliana* AHK4 (CRE1) and *M. truncatula* MtCRE1 receptor receiver domains (REC, or RD) have been published more recently [17]. We should note that dimerization of the receiver domains in the full-length receptor may be prevented by other domains. Thus, dimeric forms of AHK4_RD_, as well as ETR1_RD_ [51], may be the result of obtaining RD crystal structures in an isolated form (without other parts of the receptor) as if they were soluble proteins. Furthermore, the structures of many proteins homologous to various domains of the CK receptors have been published, which made it possible to apply the molecular modeling by homology [7,16].

Previously, the complete model of the ethylene receptor ETR1 was built by molecular modeling [56]. A scheme for the complete CK receptor was also proposed, based on the experimentally obtained structures. However, it had its drawbacks: first, transmembrane domains (TM) were represented schematically, and second, it lacked pseudoreceiver domains [57]. We have previously built models of all functional domains of StHK2–4 potato CK receptors, including SMs, HisKA, H-ATPase, pseudo-receiver, and receiver domains [16], as well as interfaces of protein–protein interactions at all stages of CK signal transduction [7].

The AlphaFold software [22], based on artificial intelligence, gave a strong impetus to modeling of full-sized proteins including CK receptor. AlphaFold 2 has already been used to construct models of potato StHK2–4 CK receptors. However, SM rather than full-length receptors were obtained, as the focus of the above study was on the active site and interaction with ligands [58]. The AlphaFold Protein Structure Database (https://alphafold.ebi.ac.uk/download, accessed on 17 October 2023) [59] contains files of predicted structures for the proteomes of 16 model organisms, including plants such as *A. thaliana*, *Oryza sativa*, and *Zea mays*. The models of full-length CK receptors from this database were recently reviewed [60]. However, CK receptors (as well as other proteins) are presented there as monomeric soluble proteins, and their structure was obviously calculated without considering both dimerization properties and localization in the membrane. The issue of orientation in the membrane is partially resolved using the TmAlphaFold database (https://tmalphafold.ttk.hu/, accessed on 17 October 2023), which is the collection of alpha-helical transmembrane proteins from the AlphaFold database. It contains structures predicted by AlphaFold2, together with information about the membrane plane, determined by TMDET algorithm, and an evaluation of structure data regarding the double lipid layer as structural constraint [61]. However, this is still not a positioning in the biologically relevant membrane, as the program only outlines the surfaces of the potential lipid bilayer. In addition, non-transmembrane regions entering improperly the hypothetical membrane remain in proteins. Finally, the receptors in this database are still monomers (Figure S10).

In studies of CK signaling, only receptor dimers are generally considered to be relevant due to cross-phosphorylation of subunits, whereas monomeric forms should therefore be inactive. At the same time, data have been reported that phosphorylation of the rice receptor OsHK6 occurs not in the *trans*-, but in the *cis*-type [62], which suggests the possibility of its activity in the monomeric form [56]. However, this conclusion needs independent confirmation. In general, this exception, if it exists, only confirms the general rule: the bulk of sensor histidine kinases function only in the form of a dimer. Therefore, our study focused on 3D models of receptor homodimers. For molecular modeling, sensor histidine kinases from two species, Arabidopsis and potato, which have been extensively studied experimentally, were selected.

### 3.2. Modeling of Full-Length CK Receptors Using AlphaFold Multimer

Using the AlphaFold Multimer software (version 1.0) [63] implemented in the COSMIC^2^ web service (https://cosmic-cryoem.org/, accessed on 5 November 2022) [64], models of full-length AHK2–4 receptor dimers were first built. It is important to note that several models were obtained for each receptor, in which the orientation of the SMs and the boundaries of their dimerization interfaces differ. In some variants, for example, SMs are oriented relative to each other in the dimer, similar to the crystal structure of AHK4 [15], and form an interaction interface in their distal parts. There were also unusual conformations in which either the mutual orientation and the distance between protomers did not suggest a reliable interaction, or the geometry of the entire receptor was completely distorted (Figure S11). The conformation in which the relative position of the dimeric SM subunits corresponds to the crystal structure of AHK4 [15] was taken as a basic one for use in further studies. The models were then optimized and embedded into the virtual artificial membrane (phosphatidyl-ethanolamine based) using the YASARA Structure software (version 22.9.24) [65] (Figure S12). The respective methods are described in detail in the Supplementary Materials.

It should be noted that the type of cell membrane holding CK receptors represents an important aspect of CK signaling. Cumulative data indicate the general divergence among membrane types in physical parameters and chemical composition [66,67,68,69]. Therefore, characteristics of the membranes should certainly be considered, along with the modeling progress. In the subcellular localization studies, CK receptors were found to reside on both endoplasmic reticulum (ER) and plasma membrane (PM) [70,71,72,73,74,75] in various proportions [76], although some authors asserted the localization of CK receptors exclusively on ER membranes [77]. The experimental pH dependencies of hormone binding by CK receptors [72,73,78] argued for the ER as a main subcellular platform for receptor functioning. The bioinformatic calculations are largely consistent with this conclusion. The electrostatic complementarity of contacting surfaces in SM dimers was found to be quite high in solutions with pH above 7 (which is typical for the ER), but significantly decreases when the medium is acidified below pH 7 (which is typical for apoplast) [7,73]. This feature provides more favorable conditions for the functioning of the CK receptors in the ER rather than in the PM. However, the receptor pool residing on the PM should not be ignored. In this study, the models built in ColabFold and discussed below were embedded in an ER-mimicking membrane.

Localization of CK receptors in both ER and PM seems reasonable in the context of the cell metabolic regulation. Exogenous CKs enter the cell via two pathways, apoplastic and/or symplastic. The apoplastic pathway leads up to the PM, whereas the symplastic pathway (through the plasmodesmata) functions via the ER [72]. Sensory parts of CK receptors embedded in the ER or the PM are exposed into ER lumens or apoplast, respectively. Such localization allows the cell to react without delay to hormones coming through any transport pathway. The ratio between ER and PM receptor pools may be indicative of predisposition of the cell to CK signals from either symplast or apoplast.

The resulting models obtained in AlphaFold Multimer confirmed most of assumptions made earlier. The first one concerns TM domains. The upstream TM domain closest to the SM at the N-terminus is rigidly fused to the α1 helix of the SM, forming a long (“pivotal”) discontinuous helix; the downstream C-terminal TM α-helix is fused to the proximal PAS domain flexibly, via a loop. Concurrently, the α1 helix of the HisKA domain is rigidly linked to this C-terminal TM domain providing its direct extension. Despite the apparent monotony of the spatial structures, various parts of the fused α-helices greatly differ in terms of the sequence conservation. A membrane-distal part of SM’s α1-helix turns out to be extremely conserved [14]; this part is responsible for the SM dimerization. A membrane-proximal part of the long α1-helix, however, seems to lack any clear consensus motif. The same applies equally to the fused TM helix, except that this kind of helix, with an average length of 21–22 residues [14], consists almost exclusively of non-polar aa. In contrast, the downstream TM domain adjacent to C-part of the CHASE domain and consisting also of hydrophobic aa, demonstrates a true consensus motif Axxx(S/A)x(G/L)x(L/F)VIx(L/F)LxG(Y/H)I [14]. This highlights the importance of downstream TM helix in signal transduction from the receptor SM to next targets in the N to C direction. Another relevant feature of TM domains is their obvious participation in subcellular localization. However, so far no signaling aa sequences for protein sorting have been detected in the primary structure of CK receptors. Therefore, it seems plausible that these transmembrane proteins are transported across the cell according to the main trafficking pathway, starting from ER membranes and after some time entering the PM through the Golgi apparatus [79].

The most important confirmation of previous assumptions concerns the domain ensemble architecture of the cytosolic part of the receptor. In the configuration in which the RD receives phosphate from the HisKA domain (the configuration that the built models possessed), the receiver and H-ATPase domains interact directly with the HisKA domain, whereas the pseudoreceiver is at the periphery of the complex and does not interact with HisKA (while interacting with the receiver and H-ATPase domains), probably regulating the function of the RD. This conformation of the cytosolic part of the CK receptor correlates with the crystal structure of the ShkA of *Caulobacter vibrioides* [39], the first hybrid histidine kinase with two RDs whose complete structure has been solved experimentally. Full-length models confirm *trans*-phosphorylation of CK receptors [29,45]. H-ATPase domain interacts with the α2-helix of the HisKA domain of the same (conditionally A) subunit and the α1-helix of its neighboring (conditionally B) subunit. The active site of H-ATPase chain A is directed toward the conserved phosphoaccepting histidine of chain B and vice versa, making cross-*trans*-phosphorylation conformationally possible. The RD of chain B in the phosphate receiving position, however, interacts with the α1 and α2 helices of the HisKA domain of the same (B) subunit.

The TM domains in the obtained models are correctly oriented relative to each other in terms of potential localization in the membrane, so that incorporation of the receptors into the membrane did not require additional interference in their structure. However, intersubunit interactions in the TM region occurs mainly through the C-terminal TM domains. The number of N-terminal TM domains corresponds to the previously predicted numbers of 1, 2, and 3 TM helices per subunit in AHK4, AHK3, and AHK2, respectively [14]. In the Alphafold Multimer AHK3 models, the region in the intracytosolic region between the two N-terminal TM helices in one version has helical folding, and in the other it is almost unfolded. In both cases, this region, while not being a transmembrane domain, is oriented in such a way that it falls into the profile of the hypothetical membrane (Figure S13).

The AHK4 and AHK2 models have fairly large unstructured areas. In AHK4, this is the N-terminal fragment up to the first TM domain, and in AHK2, in addition to the small (compared to AHK4) N-terminal fragment, there is also an extensive unfolded extracytosolic region between the first and second TM helices. (Figure S13). According to preliminary data, this site in AHK2 may be a rudimentary degenerate analog of the SM, although this did not manifest itself in the models created in AlphaFold Multimer.

Modeling in AlphaFold has several vulnerabilities. In particular, models obtained using this artificial intelligence can have areas with both high and low Model Confidence scores. AlphaFold is still template-based modeling software. Most often, the regions evaluated as less reliable correspond to those fragments of the sequence for which there are no templates—experimentally resolved structures. This is reflected in the per-residue confidence score (pLDDT) of the models (Figure S14). It can also be observed when comparing the complete structures of AHK4 with AHK2 and AHK3 published in the AlphaFold Protein Structure Database [59]. Inserts in the PAS domains of AHK2 and AHK3 that are lacking in the PAS domain of AHK4 have the lowest values. Thus, in order to obtain more accurate models that allow studying the fine mechanisms of receptor functioning, strong optimization is required. In addition, the AlphaFold Multimer failed to build a heterocomplex of the receptor dimer with HPts. The HPt subunits were simply “thrown out” by the program outside the receptor structure. This problem was solved using ColabFold, as discussed below.

### 3.3. Modeling of Full-Length CK Receptors and Their Complexes with HPts Using ColabFold

**Figure 2:**
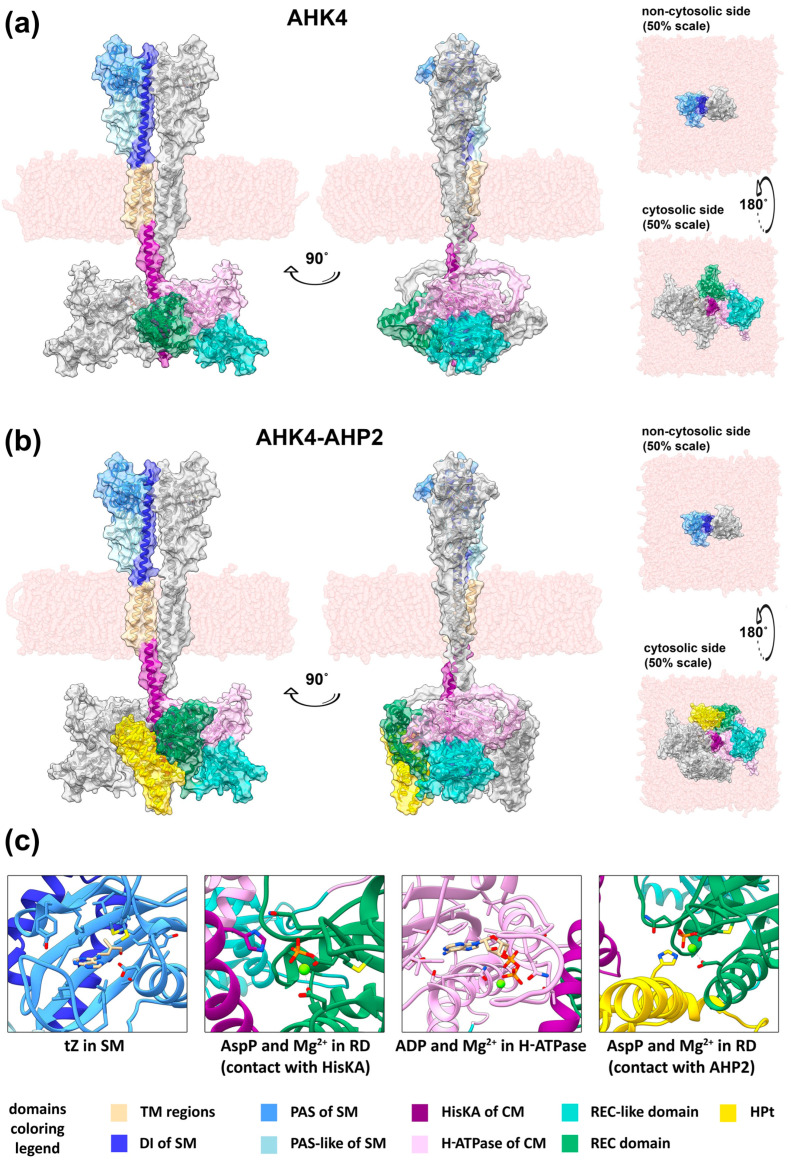
Models of the full-length CK receptors obtained using ColabFold and embedded in the membrane in the YASARA Structure software. General view of the dimer of full-length AHK4 in HPt-free form (**a**) and in complex with two AHP2 proteins (**b**), presented in ribbon view with a semitransparent surface overlay. The N-terminal fragments of AHK4 up to the first TM domain have been removed. Hydrogen atoms have also been removed. Both models are presented in four projections: “front” (left), “side” (center), “top”, i.e., looking at the non-cytosolic side (facing the ER lumen or apoplast) of the membrane (top right), and “bottom”, i.e., looking at the cytosolic side of the membrane (bottom right). The “top” and “bottom” views have a scale of 50% relative to the “front” and “side” views. (**c**) Highlighted active sites of the AHK4 dimer and the AHK4–AHP2 complex with ligands: tZ in SM; phosphoaspartate (AspP) and Mg^2+^ in RD (in contact with HisKA); ADP and Mg^2+^ in the H-ATPase domain; AspP and Mg^2+^ in RD (in contact with AHP2). The viewing angles have been changed relative to (**a**,**b**) for better visualization. The surface and hydrogen atoms have been removed.

Homodimers of *A. thaliana* AHK4 and *S. tuberosum* StHK4 receptors and their complexes with phosphotransfer proteins AHP2 and StHP1a, respectively, were constructed using ColabFold (version 1.5.2) [23], implemented in the COSMIC^2^ web service (https://cosmic-cryoem.org/, accessed on 7 August 2023) [64] (Figure 2a,b and Figure S15). In contrast to the AlphaFold multimer, there were no “unexpected” SM dimer conformations in all models generated in ColabFold (Figures S16 and S17). In addition, ColabFold did not eject HPt from the receptor when modeling the HK–HPt interaction, thus making it possible to obtain genuine complexes (Figure 2b and Figure S15). The models obtained in ColabFold were similar whether templates were used or not, so the template-based method was chosen for unification. The sequence coverage of models with templates is shown in Figure S18, and pLDDT score of models—in Figures S19 and S20. Using AlphaFill (https://alphafill.eu/, accessed on 10 August 2023) [80], ligands were added to the complexes: *trans*-zeatin in SM, ATP or ADP, and Mg^2+^ ions in H-ATPase domains and another Mg^2+^ in RD (Figure 2c). Post-translational modifications (phosphorylation) of phospho-accepting residues were carried out in ViennaPTM (http://vienna-ptm.univie.ac.at/, accessed on 14 August 2023) [81].

In total, after the addition of ligands and post-translational modifications, the following versions of AHK4 and StHK4 receptors were obtained (all with bound tZ and two Mg^2+^ ions in the RD and the H-ATPase domain): HPt-free forms with bound ATP, HPt-free forms with bound ADP and phosphorylated conserved aspartate in RD, HPt-associated forms with bound ADP and phosphoaspartate in RD. The initial unliganded forms, provided in the Supplementary Materials, are not true apo-forms, primarily because the ligand-bound form served as a template for SM. All complexes after energy minimization in YASARA software (version 22.9.24) [82,83] were tested in ProCheck implemented in PDBSum (http://www.ebi.ac.uk/thornton-srv/databases/pdbsum/, accessed on 16 August 2023) [84,85] and showed high stereochemical quality of the models (Table S1). Finally, the complexes were embedded in a membrane mimicking the lipid bilayer of the ER, with a proportion of glycerolipids and sterols roughly corresponding to that experimentally determined in the reticulum (Figure 2a,b, Figure S21 and Figure S22) [66].

The structure of individual domains in the obtained full-length receptor models, as expected, generally confirmed previous predictions [7,16], and was similar to the selected conformations obtained in AlphaFold Multimer.

In general, according to the models obtained, receptor dimers are twisted-shaped. To be more precise, in the SM and TM regions they exhibit the properties of parallel dimers, while in the cytosolic portion in the membrane-proximal part of the HisKA domain twisting occurs. Thus, from a “frontal” perspective (i.e., a view in which the receptor is seen in its entire length, the central axis is parallel to the image plane, and the SM subunits are located symmetrically to the left and right of this axis), the SM of one subunit will be located conventionally on the left, whereas the catalytic and receiver modules of the same subunit are conventionally on the right. The overall shape of the dimer can be described as dumbbell-shaped, with one broad part in the SM region, a narrow part in the TM region and the membrane-proximal “stem” of the HisKA domain, and another broad part in the rest of the cytosolic portion. The narrow (middle) part of a dimer represents ultralong α-helices bundled together in the cell membrane (Figure S15) and apparently serving as molecular levers for transmitting CK signal from the extracytosolic side of the membrane to the intracytosolic side. The length of the complexes, when measured along the axis perpendicular to the membrane surface, is approximately 215 Å for the HPt-free forms and 228 Å for the HPt-bound forms. The length and maximum “width” of the SM are about 80 Å each, and the “depth” (when viewed from a frontal perspective) is about 55 Å. The length of the transmembrane regions is approximately 40 Å each. The length of the “stem” of the HisKA domain is between 31 and 36 Å. The length of all other cytosolic portions is 60 to 62 Å in HPt-free forms and about 75 Å in HPt-bound forms. The maximum “width” of the cytosolic portion is near 140 Å, and the “depth” is 100–130 Å (Figures S21 and S22).

The folding of the insertion in the H-ATPase domain, however, still raises questions; despite a small helical region, much of it remains unfolded, which contrasts with the three-helix fold predicted previously using IntFold [16]. Overall, the structure of StHK4 was very similar to AHK4, except for a few details (Figures S15–S17, S21, and S22). For example, in the RD domain, StHK4 has a larger α3-helix and a longer α3–β4 loop. Several structural differences were also detected in the H-ATPase and RLD domains, whereas in the SM and HisKA domains the structure displayed no visible differences. The N-terminal fragments upstream to the first TM domain differ in the AHK4 and StHK4 models obtained in ColabFold. In the best AHK4 model, this region was almost entirely unfolded, whereas in the others α-helical elements were formed in the center of this segment (Figure S16). In StHK4, in most models, including the best one, almost the entire N-terminal region up to the first TM was formed into a helix (Figure S17). However, disorder prediction with the PONDR web server (Figure S23) (http://www.pondr.com/, accessed on 21 October 2023) [86], the IUPred2 web server (Figure S24) (https://iupred2a.elte.hu/, accessed on 21 October 2023) [87], and, to a certain extent, DISOPRED 3 [88] implemented in the PSIPRED server (http://bioinf.cs.ucl.ac.uk/psipred/, accessed on 30 October 2023) [89] (Figure S25), showed a high degree of disorder in AHK4 and StHK4 in the area preceding the first TM. Secondary structure predictors showed in some sense the opposite result to the modeling. According to the prediction results obtained using Quick2D web server (https://toolkit.tuebingen.mpg.de/tools/quick2d, accessed on 30 October 2023) [90], StHK4 does not have α-helices in the N-terminal region before the first TM, but has a small β-element, while in AHK4, on the contrary, several small separate α-helices are predicted (Figure S26). Structure prediction with PSIPRED method [91] in combination with membrane helix prediction using MEMSAT-SVM [92] showed a disordered region of 20 aa, a β-strand, and two α-helices up to the first TM of AHK4 (Figure S27), while a disordered region of 18 aa, a β-strand, and a single α-helical formation were predicted for StHK4 (Figure S28). Notably, in the “disputed” regions of AHK2 and AHK3, PSIPRED predicted the presence of an ordered structure (Figures S29 and S30). Thus, our decision to cut off the N-terminal parts of the receptors upstream of the first TM, for further work, was made not only for the convenience of positioning in the membrane, but also due to the uncertainty about the relevance of folding of these regions in both AlphaFold multimer and ColabFold models.

The HPt-free forms of the receptors exhibited a conformation in which RD can accept phosphate from the conserved histidine of the HisKA domain. This is clearly seen after the addition of small molecules to the model along with post-translational modification of the residues (Figure 2c).

Artificial morphing between the two receptor conformations (HPt-free and HPt-bound) was performed using UCSF Chimera software (version 1.14) (see Methods in the Supplementary Materials) [93], and the corresponding animation was created (Video S1).

The resulting HK–HPt complexes exhibit an HPt binding conformation in which RD “turns away” from HisKA domain and shifts to free its place in the overall complex of the cytosolic part of the receptor. For example, in the case of AHK4 in the HPt-free form, the area of interaction between RD and HisKA covers 546.2 Å^2^ (average value for two dimer subunits), while in the HPt-bound form it decreases to 80.1 Å^2^. Thus, HPt interacting with RD partially occupies the place where RD was in the HPt-free form, which also leads to the formation of additional interactions of HPt with the H-ATPase domain of another (relative to RD) subunit.

The position of HPt relative to RD in ColabFold models is consistent to previously modeled HPt complexes with isolated RDs [7] based on the AHK5(CKI2)–AHP2 (PDB ID: 4EUK) template structure [53]. Thus, in this conformation, RD is able to transfer phosphate to HPt, which is clearly demonstrated upon the addition of a magnesium ions and the modification of conserved aspartate to phosphoaspartate (Figure 2c).

Considering the obtained models of full-length receptor structures and experimental data obtained on sensory domains—structural homologs of SM [31,37]—we can suggest a possible change in the conformation of the receptor in the absence of ligand. All of the resulting receptor models had a crossed or twisted shape type of dimerization. That is, in a frontal view, the SM of one subunit is located, for example, on the left, twisting occurs in the HisKA region, and the H-ATPase, RLD, and RD domains of this subunit are then located on the right. Therefore, in the ligand-free state, the receptor subunits in the membrane-proximal part converge and attract pivotal helices of the HisKA domain through TM domains (possibly forming a complete TM bundle). This may lead the cytosolic parts of the subunits to move away from each other, and, as a result, to block *trans*-phosphorylation. However, this hypothesis remains to be tested in future studies.

## 4. Conclusions

Here, we present original full-length models of CK receptors in dimeric forms from two species: a complete set of *A. thaliana* AHK2–4 and *S. tuberosum* StHK4 receptors. The solved full-sized structures are in general accordance with the models of some parts of the CK receptor reported earlier [7,14,16,57]. The TM domain closest to the SM at the N-terminus forms a common structural element with the α1-helix of the DI subdomain, while the C-terminal TM similarly passes into the α1-helix of the HisKA domain without breaks. The general layout of the cytosolic part of the receptor in the model corresponds to that of the crystal structure of the full-length ShkA protein. The conformation of models of the full-length CK receptor dimers confirms *trans*-type phosphorylation. That is, the phosphate from ATP caught by binding site of H-ATPase domain of one subunit is transmitted to the conserved histidine of another subunit. RD in the phosphoacceptor position interacts with the HisKA domain of the same chain, while the pseudoreceiver is located on the periphery. Models of HPt-bound forms of full-length receptors, namely AHK4–AHP2 and StHK4–StHP1a complexes, were also generated here for the first time and compared with HPt-free forms of receptors. A type of interaction in which the HPt is incorporated into the HPt-free receptor structure by displacing the RD has been proposed (see Video S1).

Models of the dimeric form of full-length CK receptors, as well as their complexes with HPts, are published here for the first time. The modeling results are considered significant enough to draw the conclusions mentioned above. In general, the models had a quite relevant layout, providing the opportunity, for example, to fill them with ligands and incorporate them into the membrane. However, some limitations still exist. First, a number of regions of AHK4 paralogs for which no templates are available have low confidence. Second, several key conformations, such as the apo-form, have not yet been obtained. Molecular modeling clearly has limitations and cannot fully replace experimental methods. However, advances in technologies, software, and in particular artificial intelligence, combined with laboratory results, may allow us to fill the gaps in our understanding of the structural aspects of CK signaling in the near future.

Our knowledge of how cytokinins trigger receptor signaling remains very limited. Experimental data evidence for the simple mode of hormone–receptor interaction, without any cooperativity signs [30,94,95,96,97,98]. This simple interaction allowed us to propose a dynamic dose–response model of receptor functioning aimed at discriminating between a few possible modes of signal induction by cytokinin. The relevant data [50] suggested that fully liganded receptor homodimers should be certainly active, whereas the activity of partially liganded receptor is very probable but still under question. For the dynamic model description and conclusions gained, see Appendix A.

The remaining basic challenges concerning the structural aspects of CK receptor functioning that have to be addressed are exemplified below. What structural changes accompany ligand binding and what is the conformational difference between the receptor apo- and holo-form? Does the receptor dimerization depend on the ligand binding and vice versa? What is the intramolecular signal transduction mechanism from the SM to the catalytic module? Can the receptor dimer signal when only one of its two sites is occupied by hormone? How many His–Asp phosphorelay events can be triggered by a bound single CK molecule? What is the limiting step in the signaling mechanism? How does the heterodimerization of the receptor impact its signaling? What is the structural background for the receptor switch between histidine kinase and phosphatase activities? What is the role of the pseudo-receiver domain, which remains the least studied structural unit of the receptors? How does the RD switch from phosphoaccepting to phosphate donor state for HPt? What is the effect of phosphorylation and magnesium ions on the interaction between HPt and RD and what determines the direction and specificity of the phosphotransfer? Does the receptor function differently depend on membrane type (ER or PM)?

The abovementioned issues can be addressed when using the whole set of experimental data concerning CK receptors, along with the recent results of full-length CK receptor modeling. The use of methods such as normal mode analysis (NMA), morphing, molecular dynamics (MD), cryo-electron-microscopy (cryo-EM), and others opens a wide range of prospects for investigating the fundamental basis of CK signaling.

## Figures and Tables

**Figure A1:**
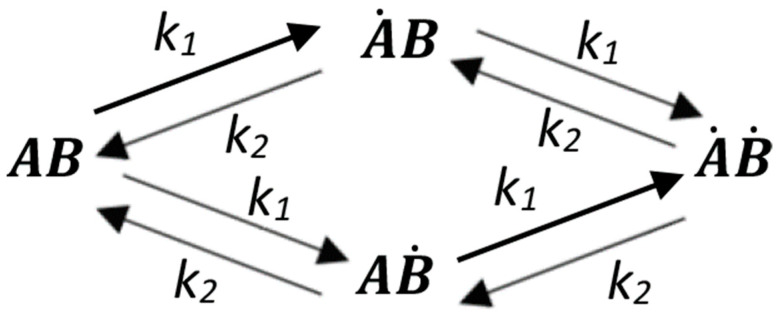
The interaction scheme of the receptor homodimer with a ligand. A and B—receptor ligand-free subunits; *Ȧ* and *Ḃ*—receptor ligand-bound subunits. *k*_1_ and *k*_2_—constants for the rate of the ligand–receptor complex formation and decay, respectively.

**Figure A2:**
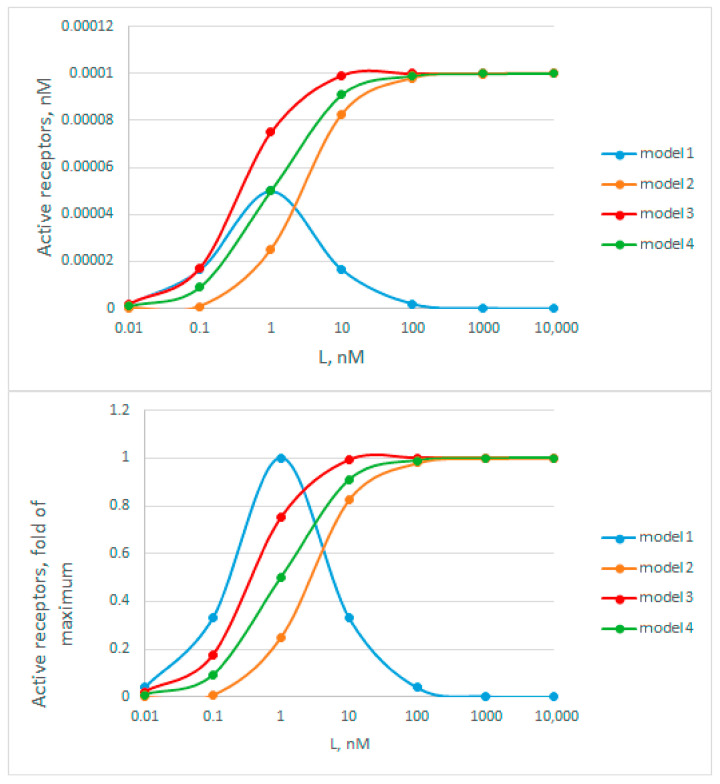
Graphical representation of receptor activity as a function of ligand concentration. The top graph shows data with absolute activity values, and the bottom graph shows relative ones. Model 1 corresponds to an active half-filled dissymmetric receptor complex, model 2—to an active fully filled receptor complex, model 3—the sum of models 1 and 2, when the receptor is active at any degree of ligand filling. Model 4—receptor activation in the absence of dimerization. For all models, Kd and *R_T_*. were taken as 1 nM and 0.0001 nM, respectively.

**Figure A3:**
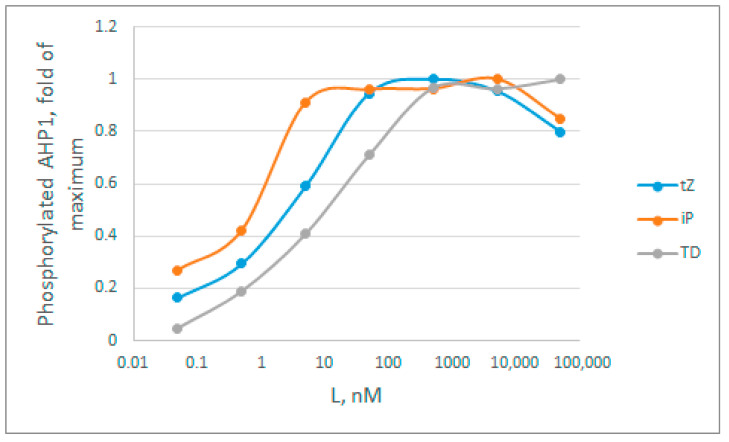
Dynamics of cytokinin-dependent phosphotransfer efficiency from the AHK4 receptor to the phosphotransfer protein AHP1, according to data from Mahonen et al. [49] (Figure S5 of the article). The intensity of ^32^P-radioactivity of the bends in SDS-PAGE was assessed using the ImageJ program. Cytokinins are designated as follows: tZ—*trans*-zeatin, iP—2-isopentenyladenine, TD—thidiazuron.

## Data Availability

Not applicable.

## Supplementary Materials

The supporting information can be downloaded at: https://www.mdpi.com/article/10.3390/ijms25010073/s1.

## Abbreviations

aa	Amino acid
CK	Cytokinin
CM	Catalytic module
MSP	Multistep phosphorelay system
HPt	Histidine containing phosphotransfer protein (phosphotransmitter)
RR	Response regulator
TCS	Two component system
SM	Sensory module
TM	Transmembrane region
HisKA (CAD)	HK-dimerization domain
RD, REC	Receiver domain
RLD	Receiver-like domain
PPI	Protein–protein interaction
DI	Dimerization interface
PM	Plasma membrane
ER	Endoplasmic reticulum
tZ	*trans*-Zeatin

## Appendix A. Simulation of the Activity Dynamics of a Receptor Homodimer Depending on Its Occupation with Ligand

The cytokinin receptor operates as a dimer, each subunit of which has a sensory module and is apparently capable of specifically binding a hormone (cytokinin). Experiments have shown that hormone binding is expressed in Scatchard coordinates as a simple linear relationship, indicating the non-cooperative nature of this binding. In this case, according to the degree of filling of vacant binding sites, a receptor homodimer with identical subunits can be in one of three states, when: (i) both binding sites are free; (ii) one of the sites is liganded, while the other not; (iii) both sites are occupied by hormones. Let us take as an axiom that in state (i), the receptor is inactive. The activation of the receptor homodimer can then occur in states (ii) or (iii) separately, or when combined, i.e., for any non-zero number of hormone molecules in binding sites.

The activation of the receptor in the cell is expressed in the triggering of signaling and response of the primary cellular target; in the case of cytokinins, this means mainly the upregulation of primary response genes. Here, we present the calculations of the dependence of the signaling degree (or induction of a response) on the dose of the hormone for all theoretically possible cases of receptor activation discussed above. The receptor subunits may be conventionally marked by the letters *A* and *B*, assuming that each subunit binds or releases the hormone regardless of the status of the other subunit of the dimer. Let us denote the rate constants for the formation and dissociation of the ligand-receptor complex as *k_1_* and *k_2_*, respectively. We can then obtain the following graphical expression of possible transitions in the hormone-receptor dimer system (Figure A1):

By the law of mass action, we can come to the following equation system for the interaction presented in Figure A1:

$$\left\{\begin{array}{c} \;\frac{dAB}{dt}=\left(2\times k_{2}\times \dot{A}B\right)-(2\;\times \;k_{1}\times AB\times L)\;\;\;\;\;\;\;\;\;\;\;\;\;\;\;\;\;\;\;\;\;\;\;\;\;\;\;\;\;\;\;\;\;\;\;\\ \frac{d\dot{A}B}{dt}={(k}_{1}\times AB\times L)+{(k}_{2}\times \dot{A}\dot{B)}-(k_{2}\times \dot{A}B)-{(k}_{1}\times \dot{A}B\times L)\\ R_{T}=AB+(2\times \dot{A}B)+\dot{A}\dot{B},\;\;\;\;\;\;\;\;\;\;\;\;\;\;\;\;\;\;\;\;\;\;\;\;\;\;\;\;\;\;\;\;\;\;\;\;\;\;\;\;\;\;\;\;\;\;\;\;\;\;\;\;\;\;\;\;\;\;\; \end{array}\right.$$

where $R_{T}$—total receptor concentration, L—ligand concentration.

Let us assume that the ligand-receptor equilibrium dissociation constant:

$$Kd=\frac{k_{2}}{k_{1}}.$$

Provided to achieve equilibrium in the system, the amounts of its components do not change and the rates of their change are, accordingly, zero. That is, if:

$$(2\times k_{2}\times \dot{A}B)-(2\;\times \;k_{1}\times AB\times L)=0$$

then we can arrive at the following relation:

$$AB=Kd\times \frac{\dot{A}B}{L}$$

Taking the latter into account, from the third equation of the above system, we can then come to the expression for the concentration of the fully liganded receptor:

$$\dot{A}\dot{B}=R_{T}-(2\times \dot{A}B)-Kd\times \frac{AB}{L}$$

Similarly, taking into account the state of equilibrium, when the change rates in the concentrations of reagents are zero, we come to the expression:

$${(k}_{1}\times AB\times L)+(k_{2}\times \dot{A}\dot{B)}-{(k}_{2}\times \dot{A}B)-{(k}_{1}\times \dot{A}B\times L)=0.$$

Substituting formulas of a ligand-free dimer *AB* and fully liganded dimer *ȦḂ* in above expressions, one can come to the formula for a half-filled receptor dimer *ȦB*. Suppose that the receptor is active only when one of the two subunits is complexed with the hormone, whereas in an empty and fully liganded states the receptor is inactive. This situation may take place when to trigger the signaling, the subunits in the dimer should not be strictly identical. Such a dissymmetry can be caused by a conformational change upon ligand binding to only one of the subunits. In this case, the amount of active receptor (*ȦB + AḂ*) would depend on the dose of the hormone (*L*) according to the following formula:

$$\dot{A}B+A\dot{\dot{B}=2\times \frac{R_{T}\times L\times Kd}{{\left(Kd+L\right)}^{2}}}\;\;\;\;\;\;\;\;\;\;\;\;model\;1$$

*ȦB* and *AḂ* states of the receptor are completely equivalent. Therefore, to correctly represent receptor activity according to model 1, these two states must be summed. Interestingly, with an infinite increase in *L*, the value of receptor activity tends to zero. At *L* = *K*d, the activity is equal to *R_T_*/2. Using this relationship, we can move on to the case of the receptor operating exclusively in a completely hormone-bound state, which is described as follows:

$$\dot{A}\dot{B}=\frac{R_{T}\times L^{2}}{{\left(Kd+L\right)}^{2}}\;\;\;\;\;\;\;\;\;\;\;\;\;\;\;\;model\;2$$

With an infinite increase in *L*, the value of receptor activity tends to *R_T_*. At *L* = *K*d, the activity will be equal to *R_T_*/4. Summing models 1 and 2 leads to the following expression:

$$\dot{A}B+A\dot{B}+\dot{A}\dot{B}=\frac{R_{T}\times L\times (2\times Kd+L)}{{\left(Kd+L\right)}^{2}}\;\;\;\;\;\;\;\;model\;3$$

With an infinite increase in *L*, the value of receptor activity tends to *R_T_*. At *L* = *K*d, the activity would be equal to three quarters of the maximum, that is, in this case, 3/4 *R_T_*. In the simplest case, in the absence of the receptor dimerization, the activity is described as follows:

$$\dot{A}=\dot{B}=\frac{R_{T}\times L}{Kd+L}\;\;\;\;\;\;\;\;\;\;\;\;\;\;\;\;model\;4$$

A graphical representation of the dependence of receptor activity on the ligand concentration (Figure A2, top) showed that in the case of the model 1, the highest activity at a ligand concentration equal to *K*d corresponds to 0.5 *R_T_*. The autoinhibition of signaling is clearly seen in this model. For other (2, 3, and 4) models, saturation is observed at *R_T_*. As for the dose efficiency of activation, on the contrary, the relative activity of the receptor increases with hormone concentrations (*L*) rising most quickly in model 1 and most slowly in model 2 (Figure A2, bottom). Interestingly, in model 3, the “acceleration” proceeds faster than in model 4. Thus, for a case of the model 3, we should expect a several (2.38)-fold decrease in the apparent binding constant (*K*d) values when estimating them at 50% signaling activity. By contrast, such *K*d estimates would be several (2.41)-fold overestimated in the case of receptor signaling, according to model 2.

To select an adequate model for receptor activation in the cell, we searched the relevant literature. As a result, a work was found, the results of which allowed us to make a choice between the above models. The work of Mähönen et al. [49] provides data on the dose dependence of the efficiency of phosphotransfer from the Arabidopsis cytokinin receptor AHK4 to the soluble phosphotransmitter AHP1. This phosphotransfer is the first link in receptor signaling and quantitatively reflects the level of its activation. In the referred work, three cytokinins were used (*trans*-zeatin, isopentenyladenine (iP), thidiazuron (TD)) in a concentration range that differed by six orders of magnitude (Figure A3). It should be noted that in this system the probable toxic effect of high concentrations of cytokinins is minimal, which makes these results more informative compared to those obtained in *in planta* experiments. Thus, these data are quite relevant for solving the problem.

By analyzing the curves in Figure A3, we immediately rejected model 1, because the curves do not have a narrow bell-shaped maximum, characteristic of this model. Next, we estimated apparent *K*d values based on the hormone concentration at which half of the activity is achieved. Here, these rough estimates are: tZ = 2 nM; iP = 0.6 nM; TD = 9 nM. According to all available data obtained in binding assays (Table S2) [30,95,98,99,100], these averaged *K*d for tZ, iP and TD are 5.1, 6.7, and 24.3 nM, respectively, which exceed the estimates from Figure A3 several times. On average, in the presented experiment, the degree of decrease in the apparent constants for tZ, iP and TD is 2.55, 11, and 2.77 times respectively, which is quite close to the theoretically calculated decrease of 2.38-fold in case of the model 3. The data on the major cytokinin tZ are particularly informative, since the *K*d value for tZ is the most reliable (Table S2). Thus, no overestimation of the constants is observed, which makes model 2 less probable. The clear underestimation of the constant values brings the cytokinin receptor signaling closer to model 3. This model has an advantage over other considered models due to its highest efficiency even when the hormone concentration is far below the dissociation constant values. However, we consider this conclusion to be tentative, and additional confirmation with more strict experimental conditions required.
