# Supplementary material for: A Model of the Full-Length Cytokinin Receptor: New Insights and Prospects
Source: Int J Mol Sci. 2023 Dec 20;25(1):73. doi: 10.3390/ijms25010073 (PMC10779265; doi:10.3390/ijms25010073)
Supplement: Supplementary file 1 [file ijms-25-00073-s001.zip › Arkhipov_ea_2023_Supplementary_Materials_revised.pdf]

Supplementary Materials

to article by Arkhipov et al. "A model of the full-length cytokinin receptor: new insights and prospects"

Methods.

Full-length structures of CK receptors were modeled in AlphaFold Multimer (version 1.0, accessed on 05 November 2022) [63] and ColabFold (version 1.5.2, accessed on 07 August 2023) [23] implemented in the COSMIC^2 web service [64] (https://cosmic-cryoem.org/, accessed on 07 August 2023). AlphaFold Multimer was used with default parameters. For the main set of models, the following ColabFold parameters were used: Number of models – 5; Number of Recycles – 3; Stop at score – 80; with the use of templates from published PDB structures; without the use of Amber molecular dynamics relaxation; max MSA depth – auto. Individual models were built without the use of templates.

The addition of ligands and ions was performed using the AlphaFill service [80] (https://alphafill.eu/, accessed on 10 August 2023). *Trans*-zeatin molecules were added from the AHK4-tZ complex of *Arabidopsis thaliana* (PDB ID: 3t4l) [15], ATP (when selected instead of ADP) and Mg^{2+} were added to the HATPase domain from ShkA of *Caulobacter vibrioides* (PDB ID: 6QRJ) [39], ADP molecules were added from the HK853-RR468 *Thermotoga maritima* complex (PDB ID: 4JAS) [40], Mg^{2+} ions were added to the receiver domain from AHK4 of *Arabidopsis thaliana* (PDB ID: 7P8D) [17]. Posttranslational modifications (addition of phosphoaspartate or phosphohistidine) were performed in the ViennaPTM 2.0 web service [81] (http://vienna-ptm.univie.ac.at/, accessed on 14 August 2023).

The models were minimized in YASARA Structure (version 22.9.24) [65] through an energy minimization experiment. To remove bumps and correct the covalent geometry, the structure was energy-minimized with the NOVA force field [82], using a 10 Å force cutoff and the Particle Mesh Ewald algorithm [83] to treat longrange electrostatic interactions. After removal of conformational stress by a short steepest descent minimization, the procedure continued by simulated annealing (timestep 2 fs, atom velocities scaled down by 0.9 every 10th step) until convergence was reached, i.e. the energy improved by less than 0.05 kJ/mol per atom during 200 steps. Quality checks of the models after minimization were performed in PROCHECK [84] implemented in PDBSum [85] (http://www.ebi.ac.uk/thornton-srv/databases/pdbsum/, accessed on 16 August 2023).

Receptor embedding into the membrane was also performed in YASARA Structure software (version 22.9.24) [65]. The following proportion of membrane components was used to mimic the ER membrane (for ColabFold models): 27% of phosphatidyl-ethanolamine (PEA or PE), 50% of phosphatidyl-choline (PCH or PC, also known as POPC), 3% of phosphatidyl-serine (PSE or PS), 7% of phosphatidyl-glycerol (PGL of PG) and 13% of cholesterol (CLR) for both layers of lipid bilayer according to [66], values were rounded.

Morphing between conformations and creating appropriate animations was performed in UCSF Chimera (version 1.14) [93]. Corkscrew interpolation method with linear interpolation rate was chosen; number of interpolation steps was 600; core fraction was set equal to 0.5. Chimera was also used to visualize models and create illustrations.

Disorder prediction was performed with PONDR web-server using all five predictors (VLXT, XL1_XT, CAN_XT, VL3-BA and VSL2) [86] (http://www.pondr.com/, accessed on 21 October 2023), IUPred2 web-server [87] (https://iupred2a.elte.hu/, accessed on 21 October 2023) and DISOPRED 3 software [88] implemented in PSIPRED server [89] (http://bioinf.cs.ucl.ac.uk/psipred/, accessed on 30 October 2023).

Secondary structure predicton was performed in Quick2D web-server with nr90 database selected and 3 MSA generation steps [90] (https://toolkit.tuebingen.mpg.de/tools/quick2d, accessed on 30 October 2023) and also with PSIPRED method [91] in combination with membrane helix prediction using MEMSAT-SVM [92] using PSIPRED server [89] (http://bioinf.cs.ucl.ac.uk/psipred/, accessed on 30 October 2023).

The images of membrane embeded forms of AlphaFold DB models were obtained using TmAlphaFold database website [61] (https://tmalphafold.ttk.hu/, accessed on 17 October 2023).

Table S1. Main Ramachandran plot parameters of modeled structures upon minimization procedures.

Complex name	Most favoured regions	Additional allowed regions	Generously allowed regions	Disallowed regions
AHK4 (unliganded)	92.3%	7.4%	0.1%	0.2%
AHK4-AHP2 (unliganded)	93.3%	6.4%	0.1%	0.2%
StHK4 (unliganded)	92.9%	6.9%	0.0%	0.1%
StHK4-StHP1a (unliganded)	93.3%	6.5%	0.1%	0.1%
$AHK4_{(AspP)}$ (tZ-ADP-Mg^{2+}-Mg^{2+})	92.5%	7.1%	0.1%	0.2%
$AHK4_{(AspP)}$-AHP2 (tZ-ADP-Mg^{2+}-Mg^{2+})	93.3%	6.4%	0.1%	0.2%
AHK4 (tZ-ADP-Mg^{2+}-Mg^{2+})	92.4%	7.2%	0.2%	0.2%
AHK4 (tZ-ATP-Mg^{2+}-Mg^{2+})	92.3%	7.2%	0.2%	0.2%
AHK4-AHP2 (tZ-ADP-Mg^{2+}-Mg^{2+})	93.3%	6.4%	0.1%	0.2%
$StHK4_{(AspP)}$ (tZ-ADP-Mg^{2+}-Mg^{2+})	93.1%	6.8%	0.0%	0.1%
$StHK4_{(AspP)}$-StHP1a (tZ-ADP-Mg^{2+}-Mg^{2+})	93.3%	6.6%	0.1%	0.1%
StHK4 (tZ-ADP- Mg^{2+}-Mg^{2+})	93.0%	6.9%	0.0%	0.1%
StHK4 (tZ-ATP-Mg^{2+}-Mg^{2+})	93.2%	6.7%	0.0%	0.1%
StHK4-StHP1a (tZ-ADP-Mg^{2+}-Mg^{2+})	93.4%	6.4%	0.1%	0.1%

Table S2. Experimental data on dissociation constants of AHK4 with different CKs.

Source	*K*d (nM) of AHK4 with cytokinins: tZ	iP	TD	References
Yamada et al., 2001	–	4.55	–	[98]
Romanov et al., 2005	2.5	–	–	[99]
	6.0	–	–	
Romanov et al., 2006	3.9	17	40	[30]
Stolz et al., 2011	4.4	3.5	–	[95]
	9.1	2.0	20	
Lomin, 2022	4.6	6.3	13	[100]
Mean values	5.08	6.67	24.33	
± SD	2.27	5.98	14.01	

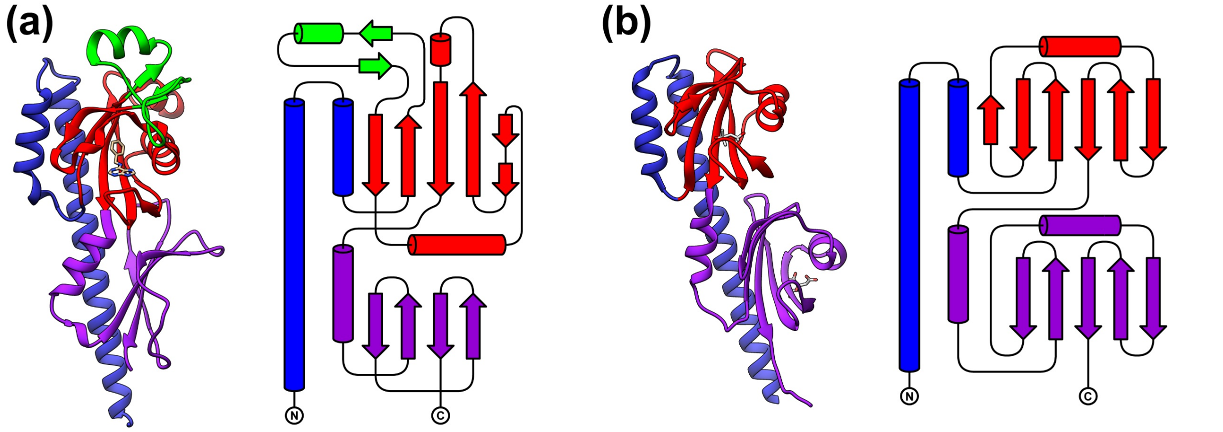

Figure S1. Structure and topology of the SMs. **(a)** Spatial structure of *Arabidopsis thaliana* CRE1/AHK4 bound to *N*6-benzyladenine (BA). (PDB ID: 3T4K) [15] and its topological scheme [14]; **(b)** Crystal structure of the Tlp3 *Campylobacter jejuni* chemoreceptor sensor domain (CcmL) (PDB ID: 4XMQ) [20] and its topological scheme. The α-helices that form the dimerization interface are marked blue. The ligand-binding PAS domain is marked red. Green is the "upper" region of the CRE1/AHK4 ligand-binding PAS domain. Purple indicates the PAS-like domain.

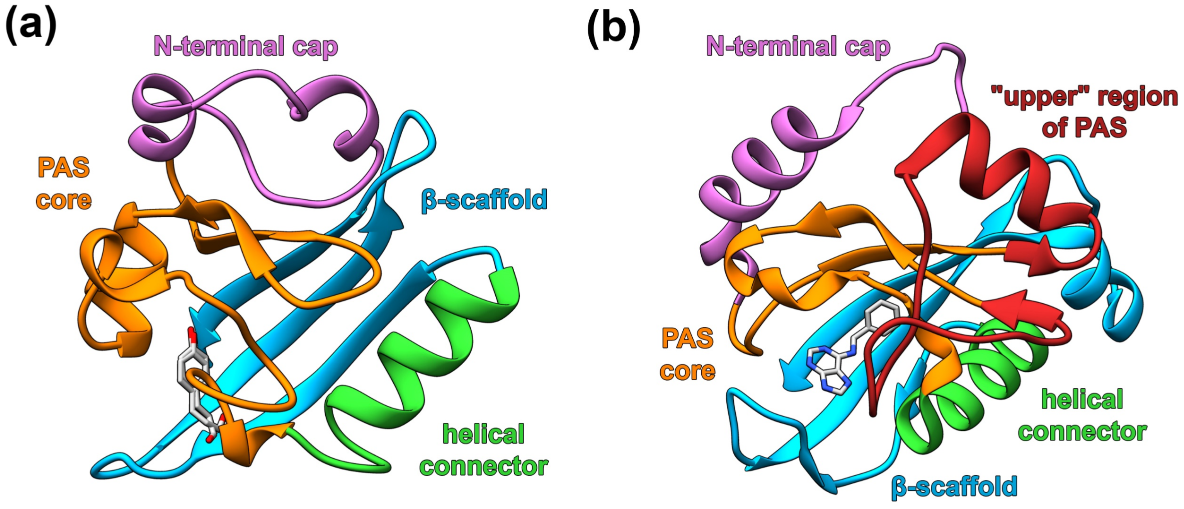

Figure S2. PAS 3D fold divided into functional parts. **(a)** *Halorhodospira halophila* photoactive yellow protein (PYP) structure in complex with 4'-hydroxycinnamic acid (PDB ID: 3PYP) [26]; **(b)** *Arabidopsis thaliana* AHK4 PAS-domain bound to BA (α1-helix and proximal PAS-like domain of SM are not shown) (PDB ID: 3T4K) [15]. Purple, N-terminal cap (which is also an α2-helix of dimerization interface (DI) in case of AHK4); orange, PAS core; green, helical connector; blue, β-scaffold; red, the "upper" region of the CRE1/AHK4 ligand-binding PAS domain [14,27,28].

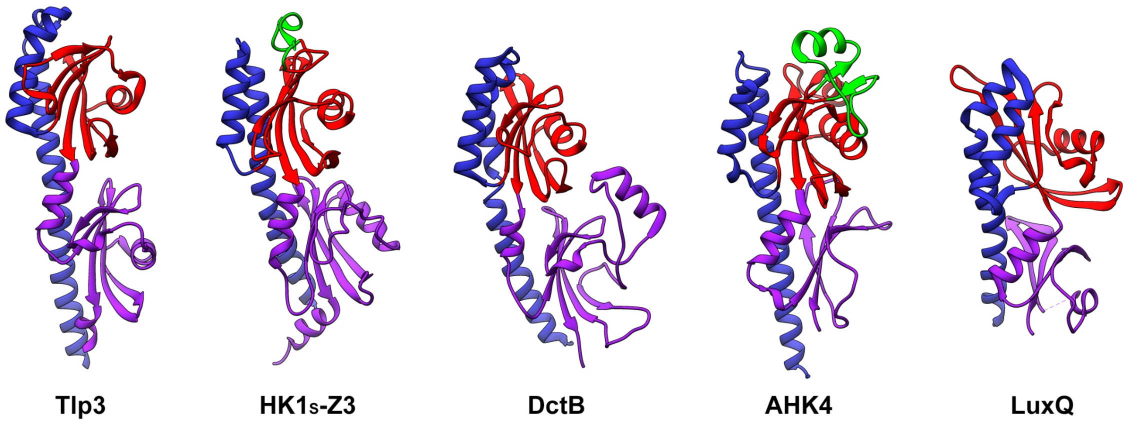

Figure S3. Similarity of spatial structures of different sensor domains (modules): *Campylobacter jejuni* Tlp3 (PDB ID: 4XMQ) [20], *Methanosarcina mazei* MmHK1s-Z3 (PDB ID: 3LIB) [31], *Vibrio cholerae* DctB (PDB ID: 3BY9) [37], *Arabidopsis thaliana* AHK4 (PDB ID: 3T4K) [15] and *Vibrio harveyi* LuxQ (PDB ID: 1ZHH) [34]. The color scheme corresponds to Figure S1.

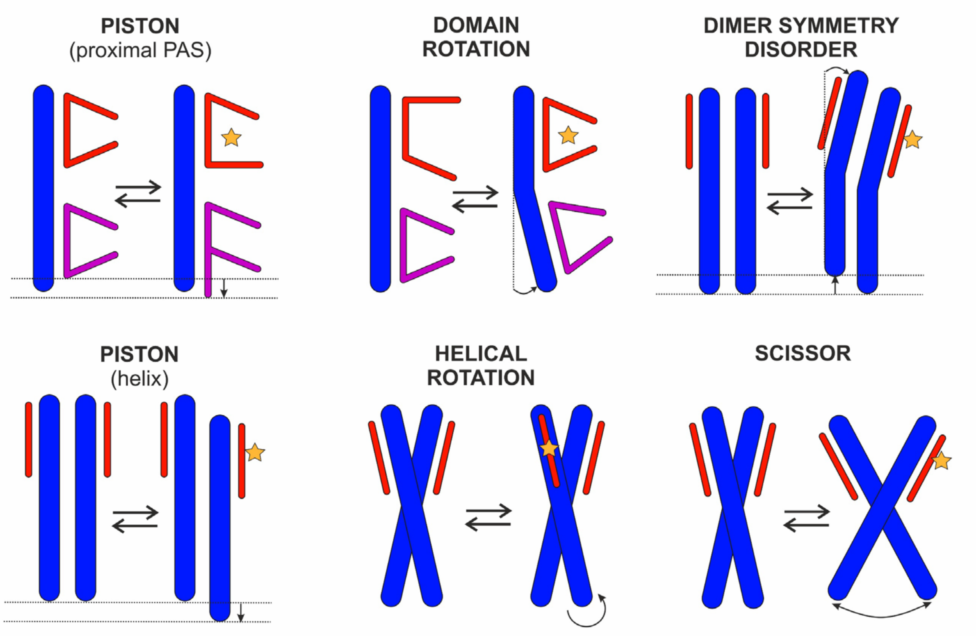

Figure S4. Conformational changes associated with ligand binding [31,32]. Above, scheme of the piston-like movement seen in comparing apo mmHK1s-Z3 and Bistris-complexed mmHK1s-Z2 from *Methanosarcina mazei* (left); scheme of the domain rotation seen in comparing apo and phosphate-complexed VpHK1S-Z8 from *Vibrio parahaemolyticus* (center); scheme of a rotation-induced translation due to asymmetry (right) [31]. Below, the piston model (left), based on the *Salmonella enterica (Salmonella typhimurium)* chemoreceptor Tar [33], helical rotation model (middle) based on the *Vibrio harveyi* LuxP-LuxQ complex [34]; the scissor-blade model (right) based on sensor domains of the *Bacteroides thetaiotaomicron* heparin binding hybrid kinase BT4663 [35] and the *Escherichia coli* magnesium-sensitive PhoQ [36]. The ligand is shown as a yellow star.

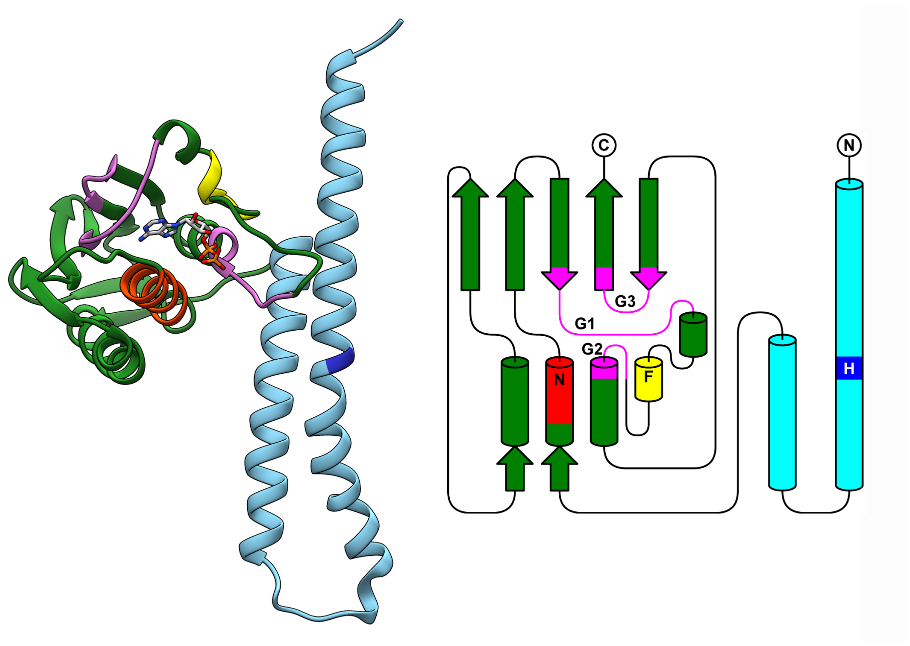

Figure S5. Crystal structure of *Thermotoga maritima* HK853 (PDB: 2C2A) [41] (left) and its topology diagram (right). The H-ATPase (CAD) domain is colored green and the HisKA (DHpD) domain is colored blue. Highlighted regions correspond to conserved boxes [42].

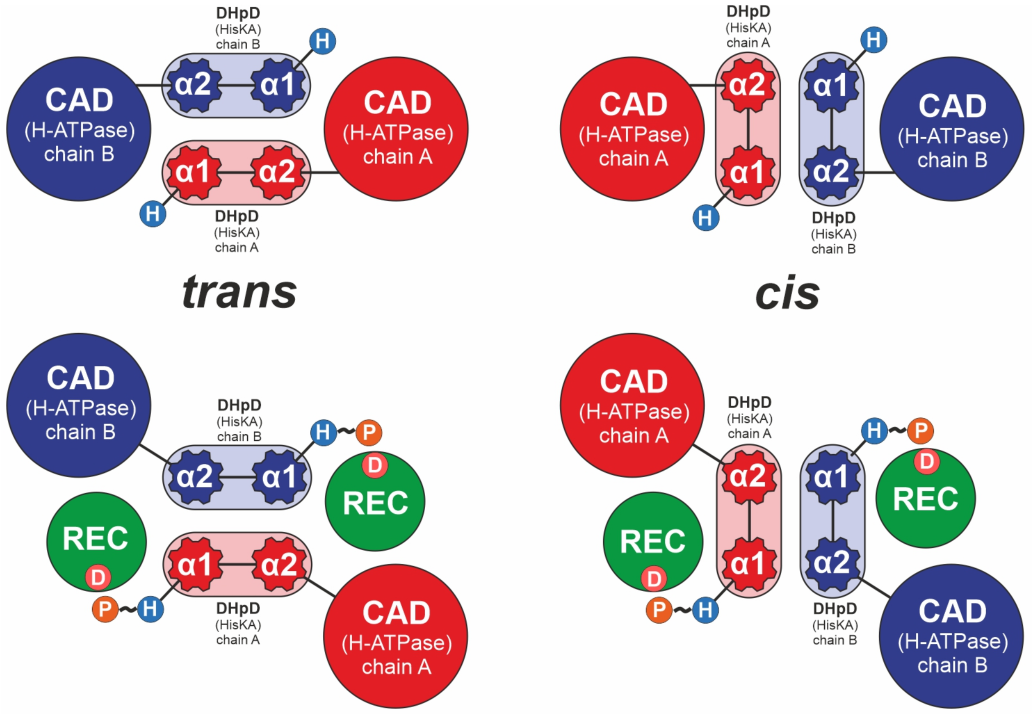

Figure S6. Schemes of *trans*- and *cis*-phosphorylation of histidine kinases [46]. Above, DHp dimers formed between chains with different loop orientations, which determine *trans*- (left) or *cis*- (right) phosphorylation. Below, interaction of the response regulator with the dimer of the DHp kinase domain during *trans*- (left) or *cis*- (right) phosphorylation. $\alpha1$ and $\alpha2$ means α-helices of DHp (HisKA) domains.

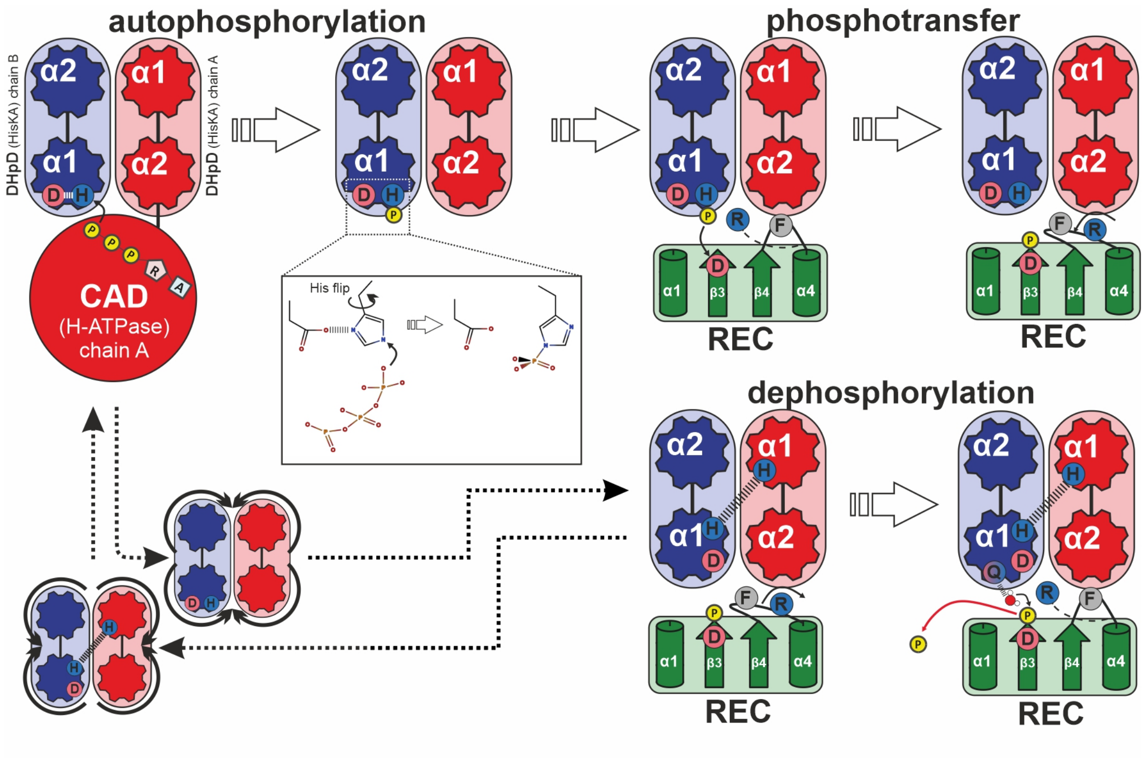

Figure S7. Conceptual model of unidirectional signaling in TCS [48]. Above, a model of autophosphorylation and phosphotransfer. Below is a dephosphorylation model. The autophosphorylation reaction scheme with His flip is highlighted. *α* means *α*-helices, β means β-strands.

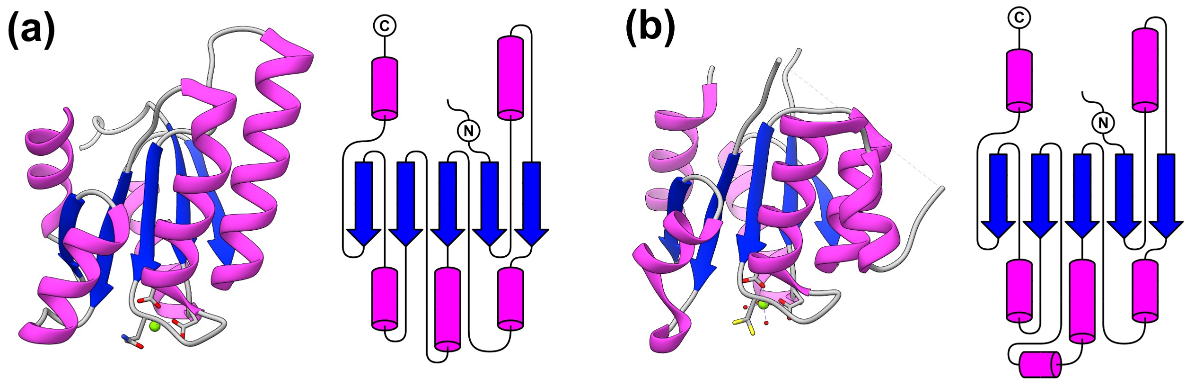

Figure S8. Structure of the RDs. **(a)** Spatial structure (left) and topological diagram (right) of *Arabidopsis thaliana* histidine kinase CKI1 RD (PDB ID: 3MMN) [52]; **(b)** Spatial structure (left) and topological diagram (right) of *Arabidopsis thaliana* histidine kinase AHK5 (CKI2) RD (PDB ID: 4EUK) [53]. Small green spheres designate Mg^{2+} ions.

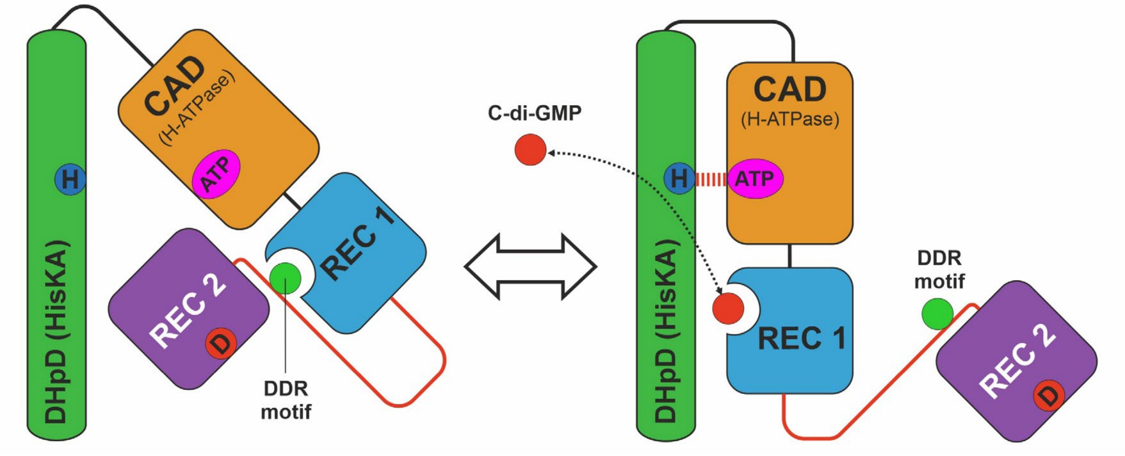

Figure S9. *Caulobacter vibrioides* ShkA activation model via c-di-GMP-induced change in conformational equilibrium [39]. C-di-GMP (red circle) competes with the DDR motif (light green circle) for Rec1 binding, resulting in Rec2 release, providing catalysis (e.g., autophosphorylation as indicated).

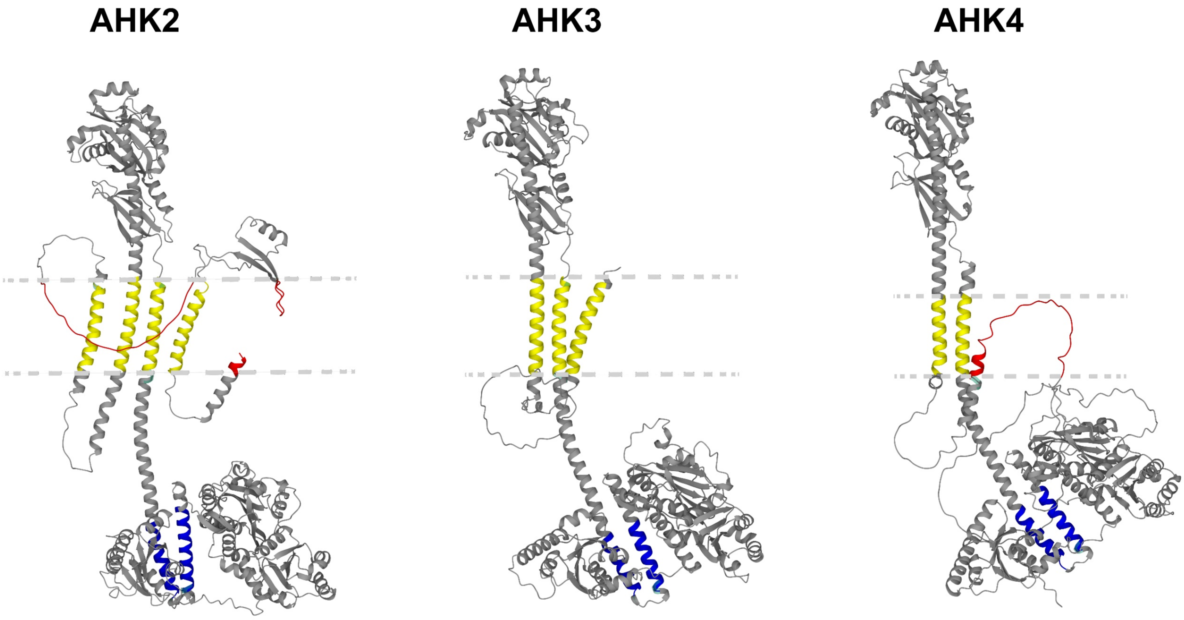

Figure S10. Membrane embeded forms and evaluation of AlphaFold models of AHK2-4. The images of the models were obtained using TmAlphaFold database [61], which contains the models predicted by AlphaFold2 [22] (and published originally in the AlphaFold database [59]), information about the membrane plane determined by the TMDET algorithm, and an evaluation of structural data concerning the double lipid layer as a structural constraint. The presented models of AHK2, AHK3 and AHK4 receptors have the following IDs in the specified databases: Q9C5U2, Q9C5U1 and Q9C5U0 in AlphaFold DB; AHK2_ARATH, AHK3_ARATH and AHK4_ARATH in TmAlphaFold DB, respectively. Transmembrane segments colored yellow; non-transmembrane regions in membrane colored red; transmembrane regions in non-membrane regions colored blue.

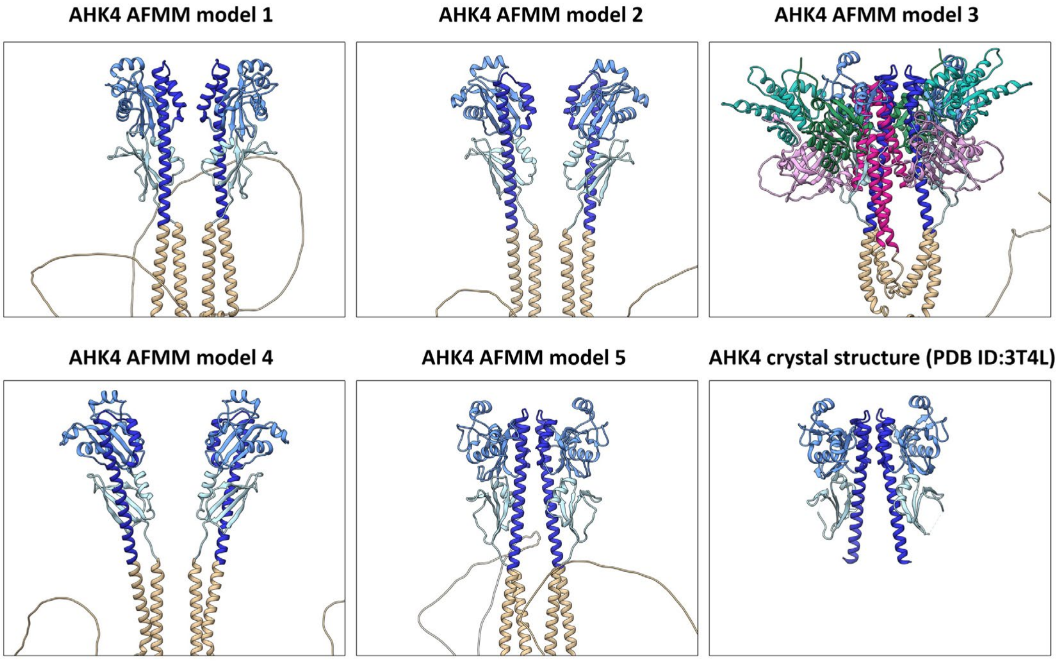

Figure S11. Top 5 models generated in AlphaFold Multimer (AFMM) [63] compared to the crystal structure of AHK4 (PDB ID: 3T4K) [15]. It is clearly visible that the conformation of the dimer in the SM region corresponds to the crystal structure only in model 5, which was chosen for further work. In models 2 and 4, the DI subdomains are too far apart to interact confidently, whereas in model 1 the subunits are rotated so that only the α2-helix of DI can participate in interface formation. In model 3, although the SM follows the outline of the AHK4 crystal structure, the overall geometry of the model is completely disturbed, due to the fact that the cytosolic part of the receptor is twisted and interferes with the extracytosolic SM. The color scheme corresponds to Figure 2 from the main body of the article.

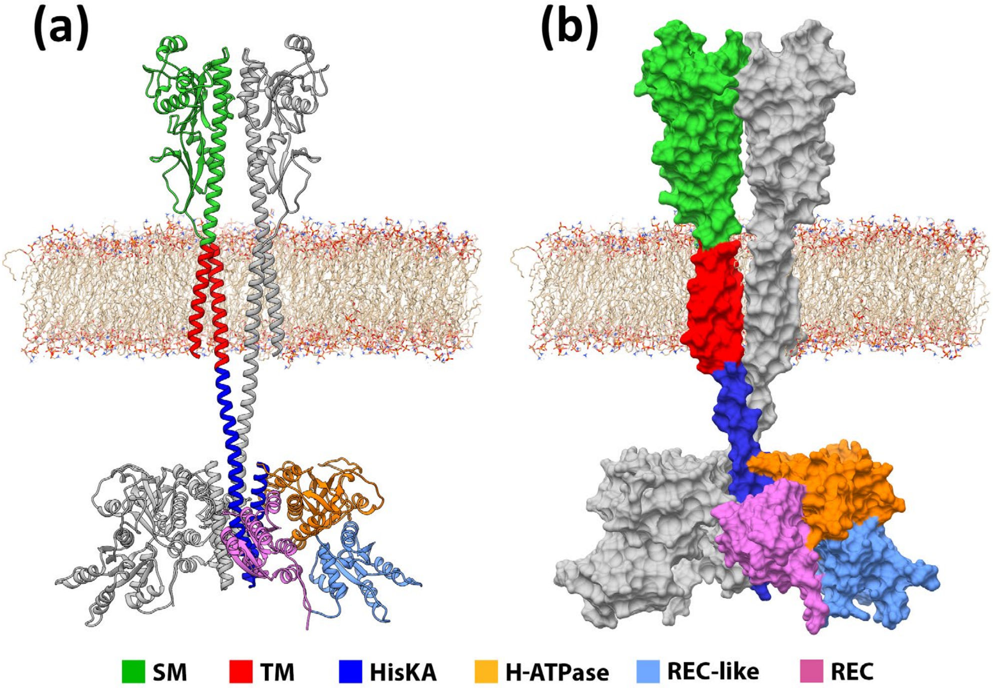

Figure S12. Model of the full-length CK receptor AHK4 obtained using AlphaFold Multimer [63] and embedded in the membrane in the YASARA Structure software [65]. **(a)** Ribbon display style; **(b)** Molecular surface of the receptor. The color codes for domains are shown at the bottom of the illustration. The N-terminal fragments up to the first TM domain have been removed.

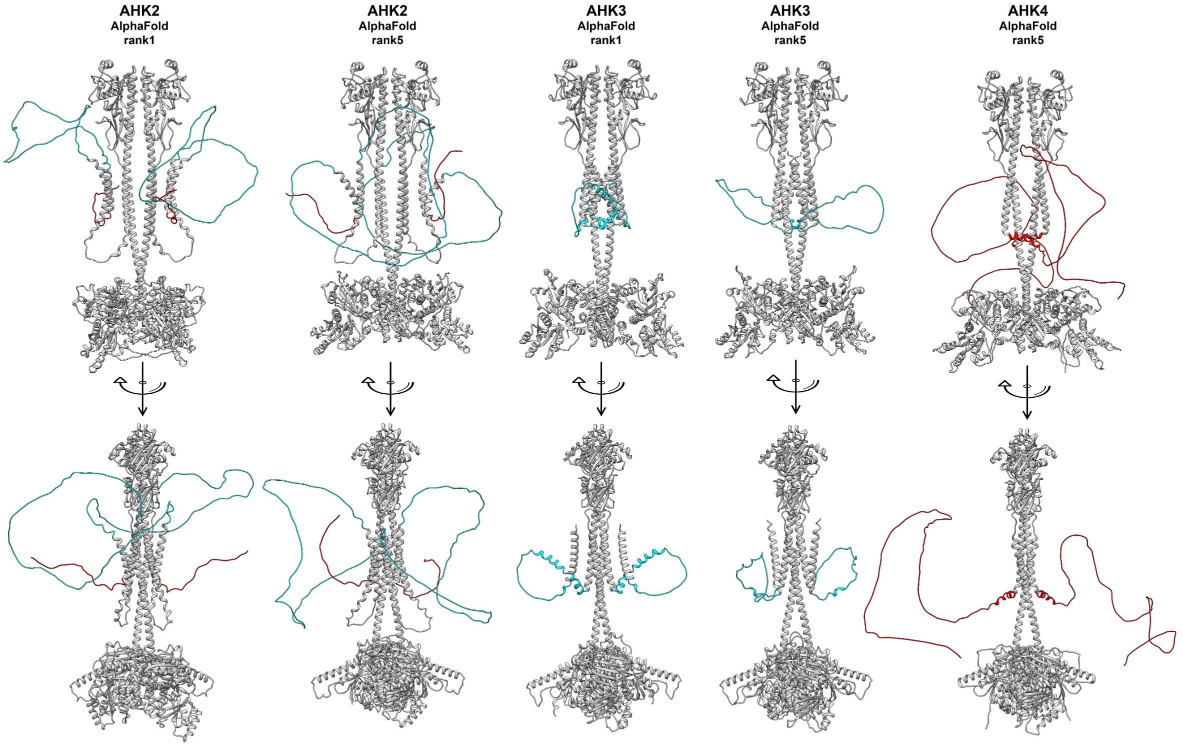

Figure S13. Uncut models of the full-length CK receptors *Arabidopsis thaliana* AHK2-4 dimers obtained using AlphaFold Multimer [63], presented in ribbon view in two projections. The N-terminal parts of the proteins up to the first transmembrane domain are not removed in this figure and are colored red. The regions between the first two N-terminal TM domains are colored cyan.

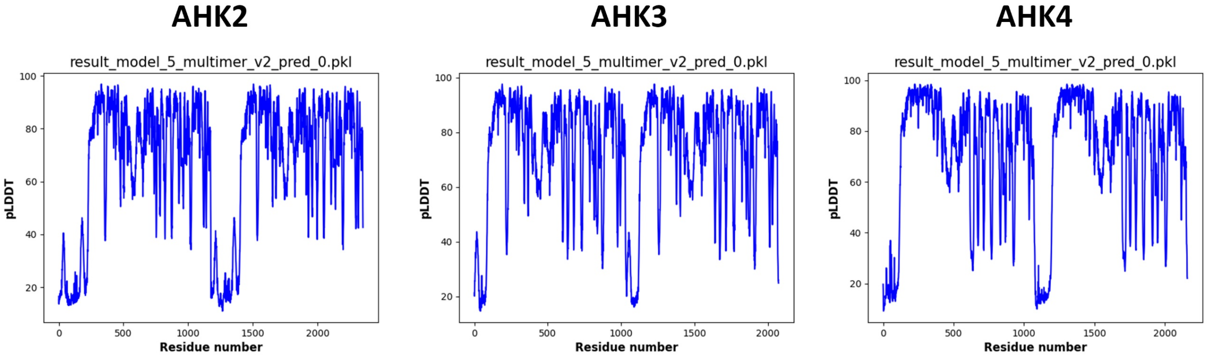

Figure S14. Per-residue confidence score (pLDDT) of models obtained in AlphaFold Multimer [63].

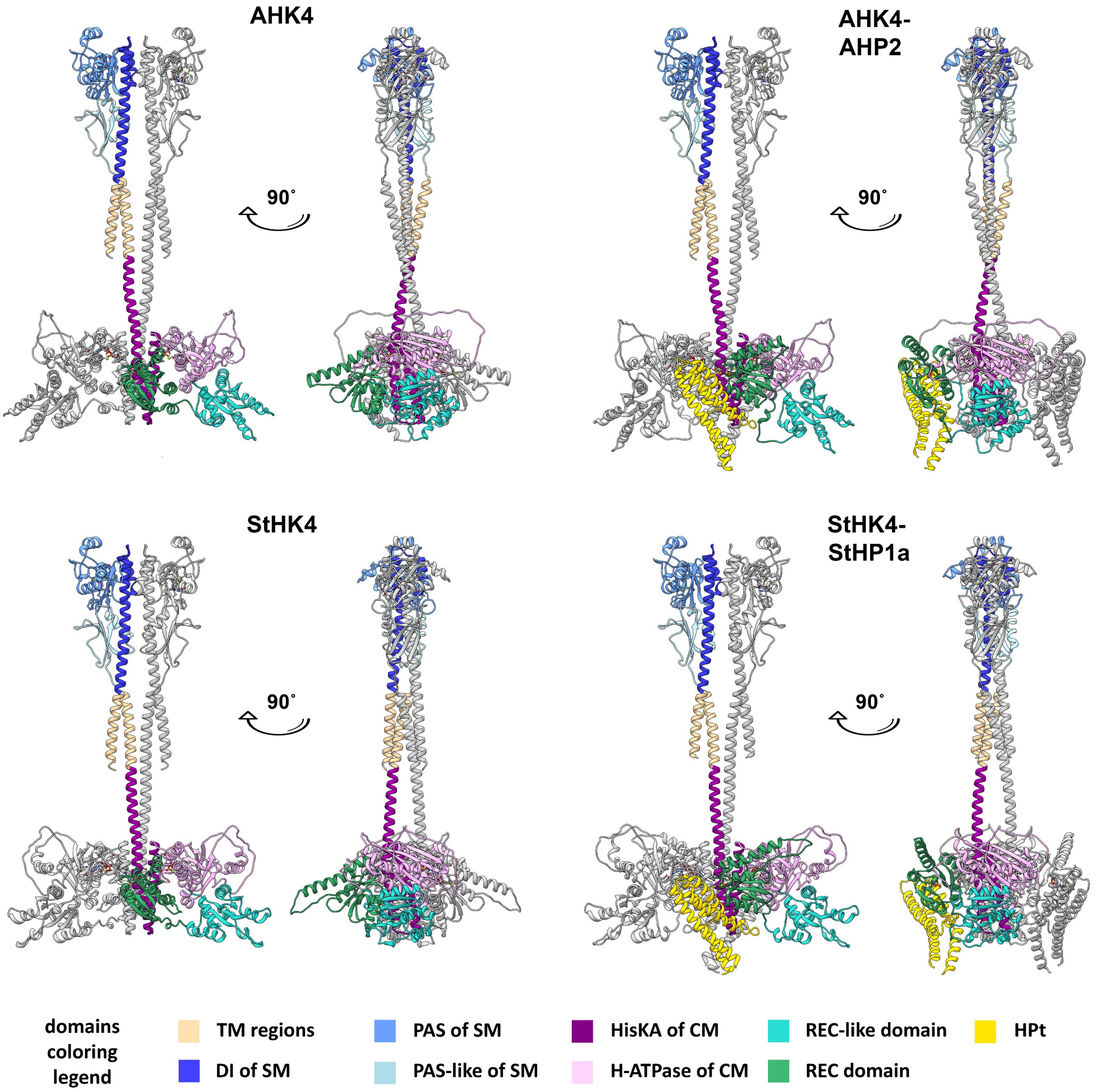

Figure S15. Best models of the full-length CK receptors obtained using ColabFold [23]. General view of the dimer of full-length *Arabidopsis thaliana* AHK4 (top) and *Solanum tuberosum* StHK4 (down) in HPt-free form (left) and in complex with two AHP2 proteins (right), presented in ribbon view. Hydrogen atoms have been removed. All models are presented in two projections: "front" and "side" (center). The color codes for domains are shown at the bottom of the illustration. The N-terminal fragments of CK receptors up to the first TM domain have been removed.

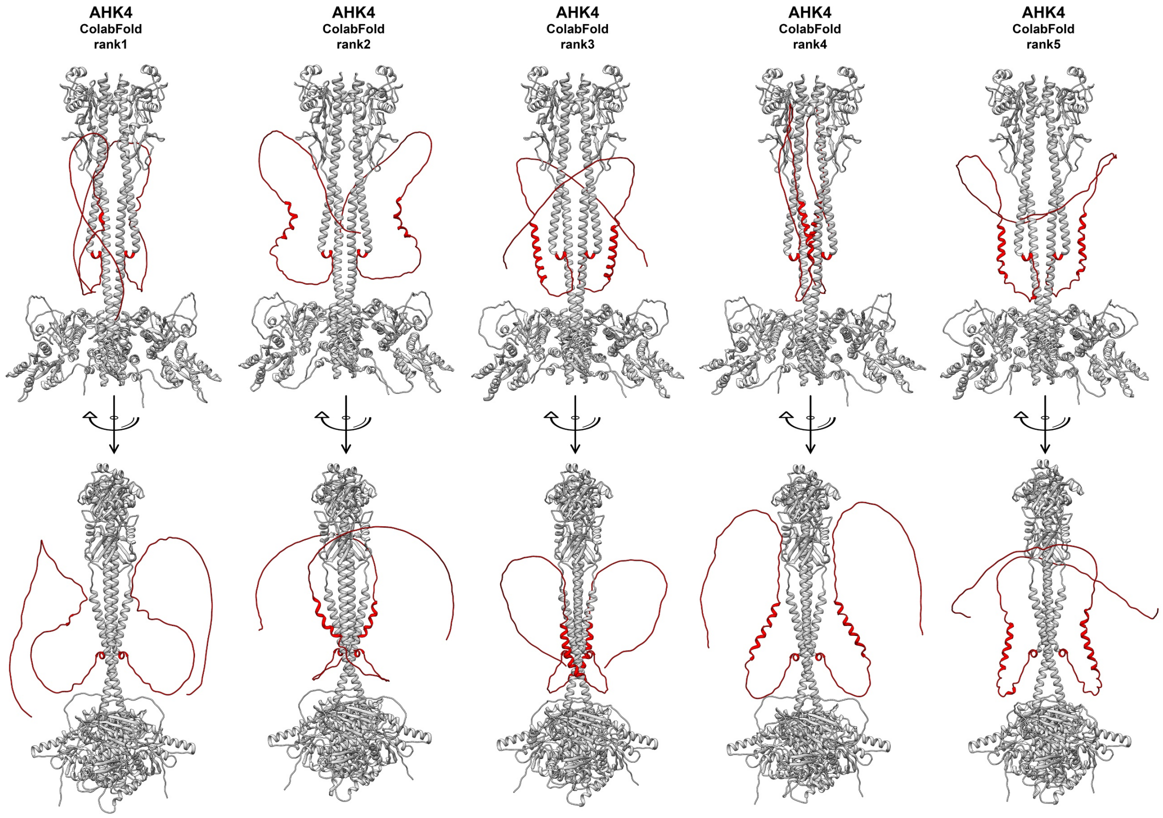

Figure S16. Uncut models of the full-length CK receptor *Arabidopsis thaliana* AHK4 dimers obtained using ColabFold [23], presented in ribbon view in two projections. The N-terminal parts of the proteins up to the first transmembrane domain are not removed in this figure and are colored red. The top 5 models according to the program ranking are shown (the best model selected for further research and presented in the main figures is rank 1)

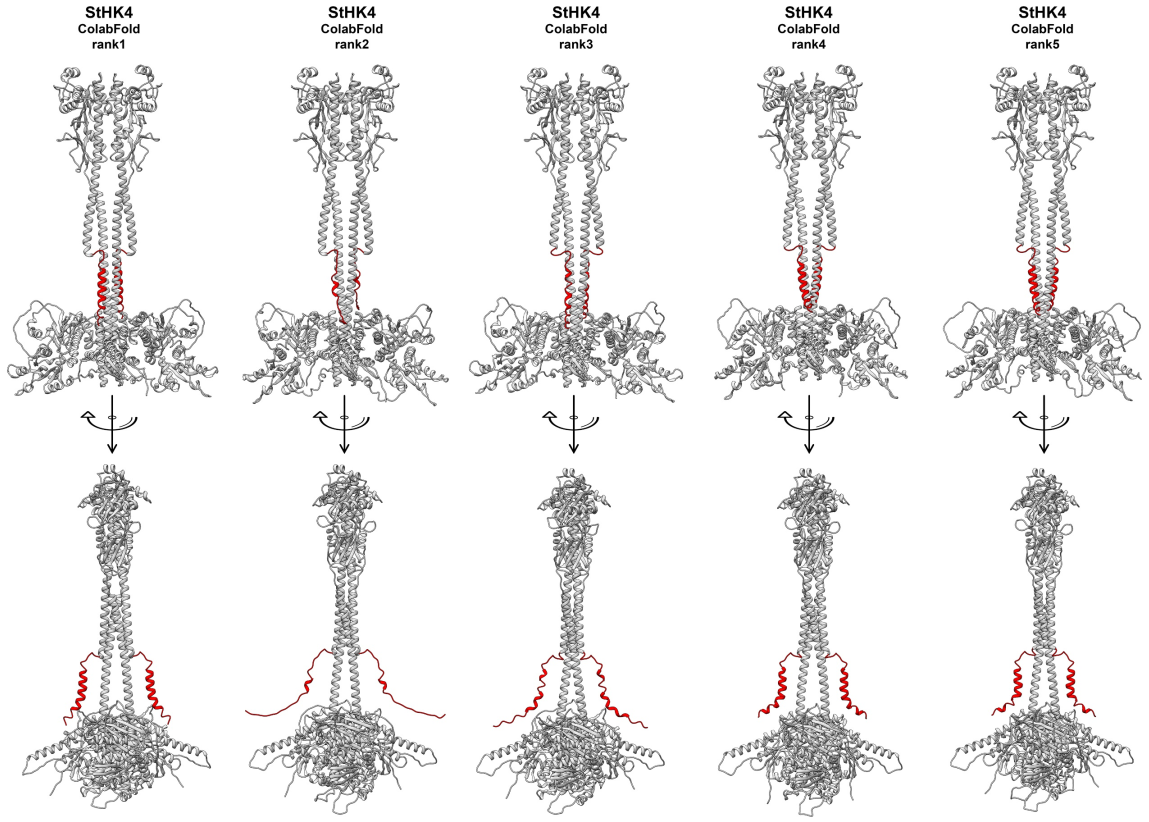

Figure S17. Uncut models of the full-length *Solanum tuberosum* CK receptor StHK4 dimers obtained using ColabFold [23], presented in ribbon view in two projections. The N-terminal parts of the proteins up to the first transmembrane domain are not removed in this figure and are colored red. The top 5 models according to the program ranking are shown (the best model selected for further research and presented in the main figures is rank 1)

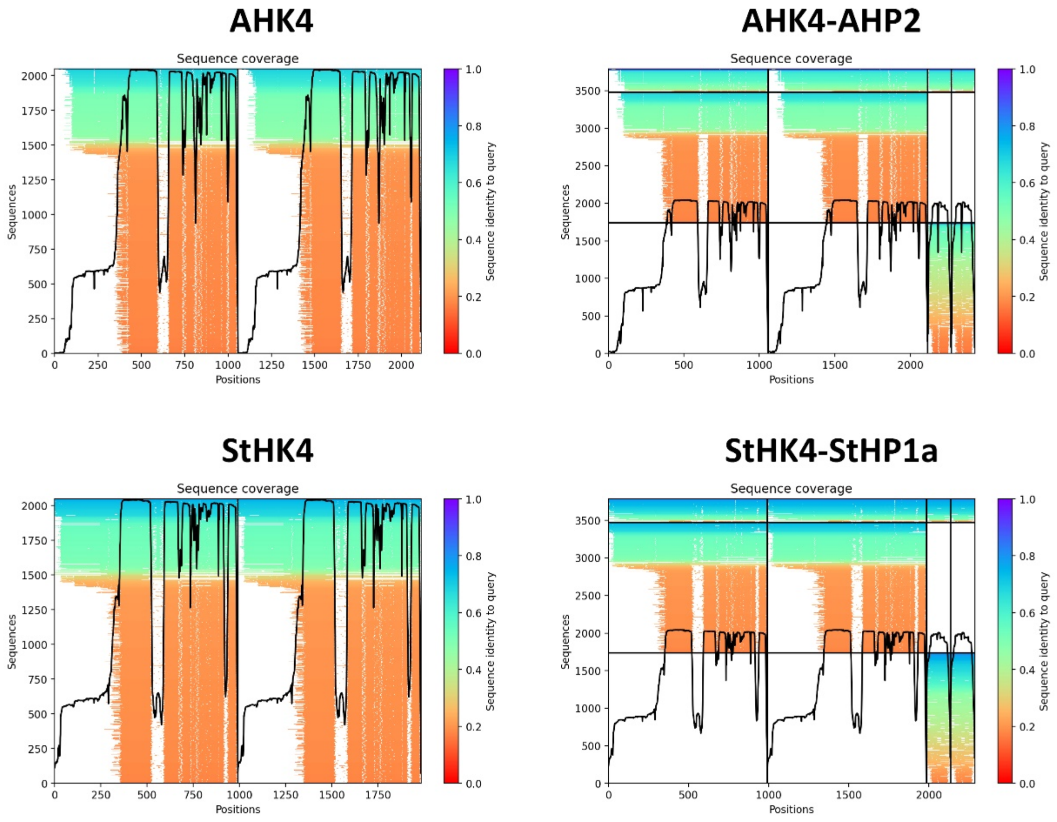

Figure S18. Sequence coverage with templates for models obtained in ColabFold [23].

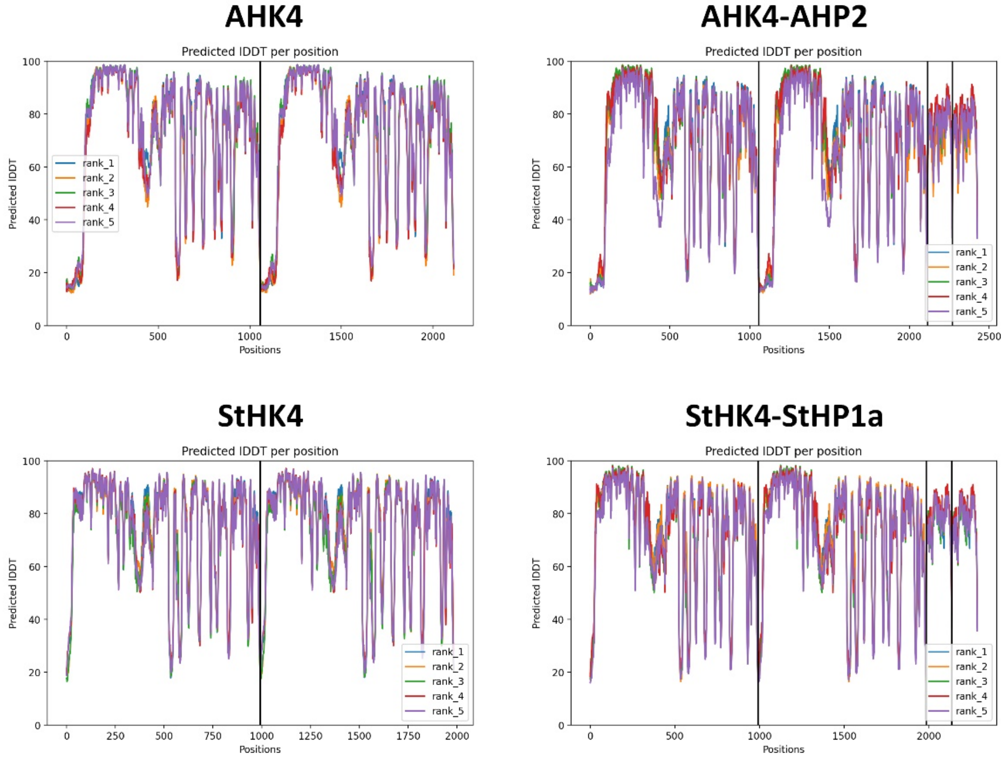

Figure S19. Per-residue confidence score (pLDDT) of models obtained in ColabFold [23].

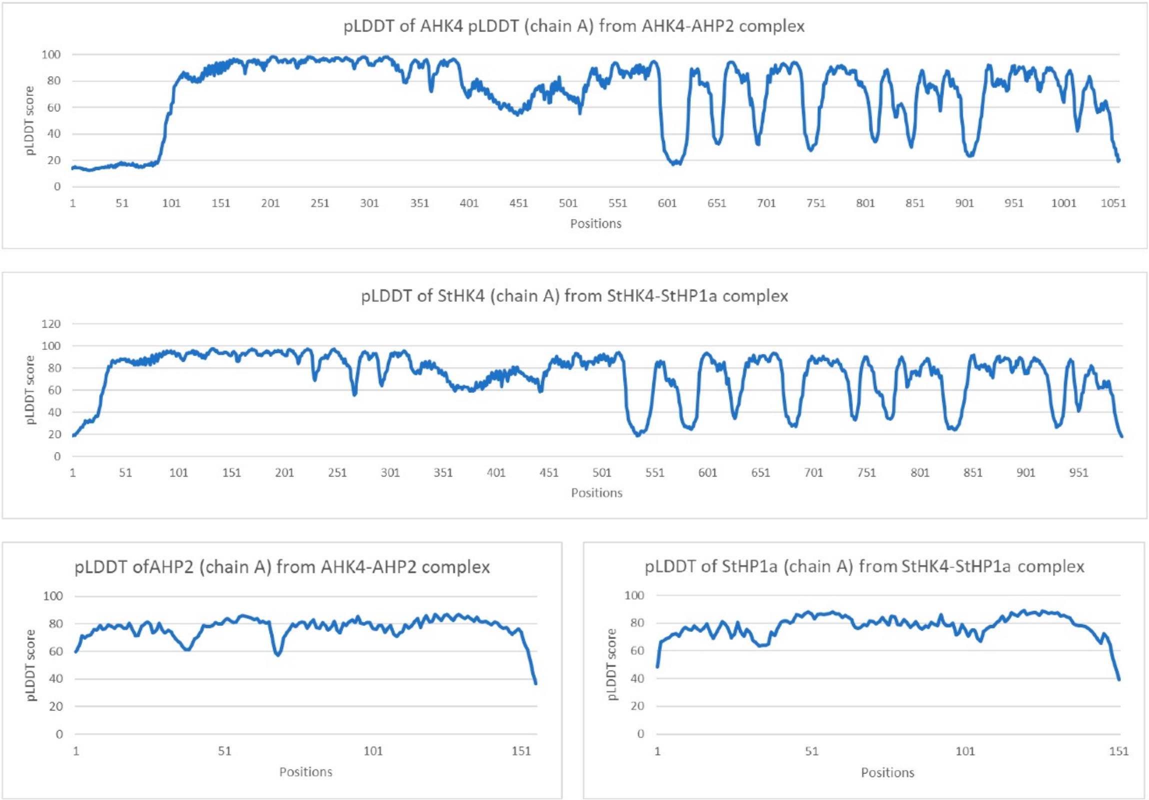

Figure S20. Per-residue confidence score (pLDDT) of models obtained in ColabFold [23]. Scaled plots for selected (rank 1) models.

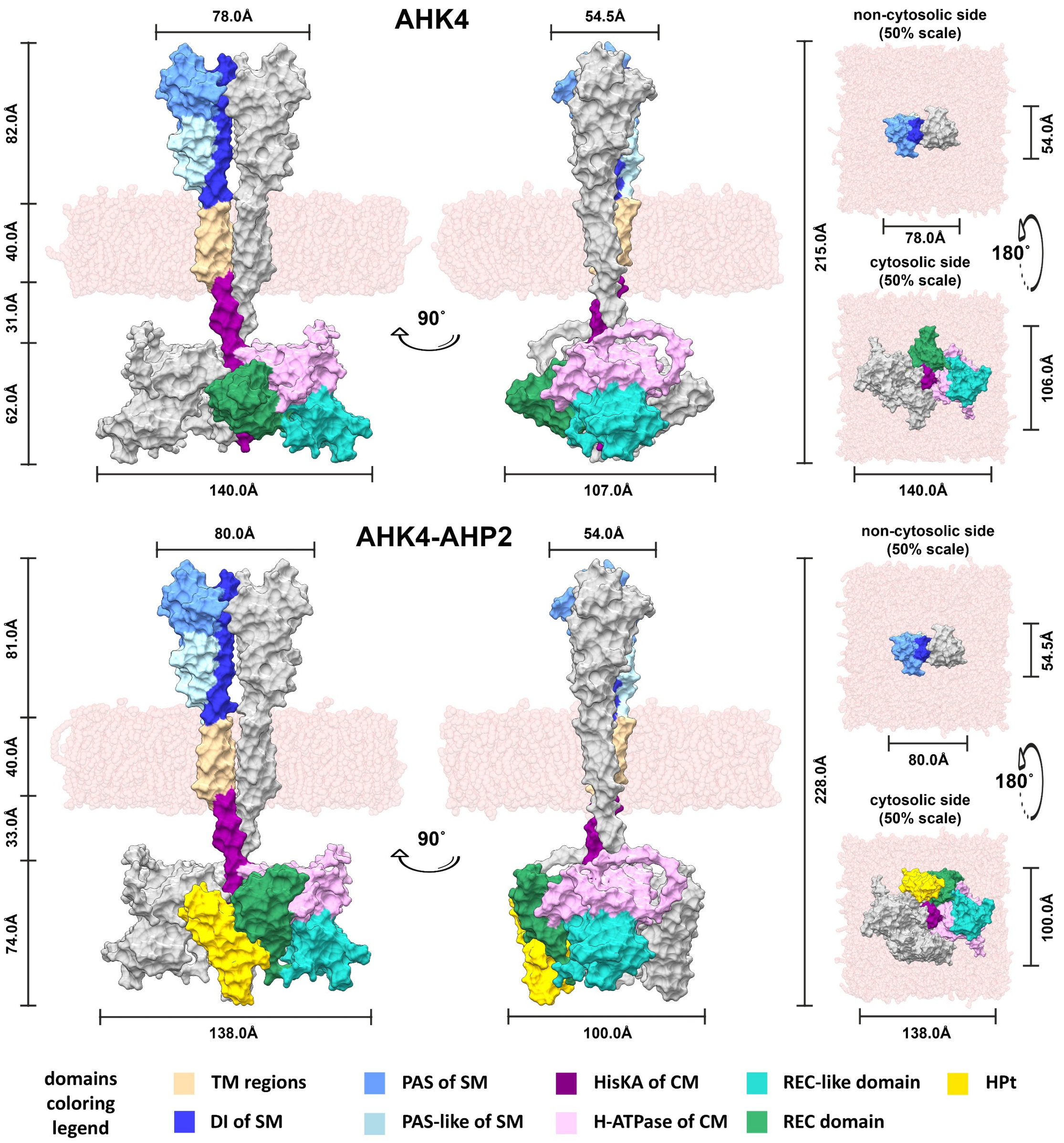

Figure S21. Models of the full-length *Arabidopsis thaliana* AHK4 receptor obtained using ColabFold [23] and embedded in the membrane in the YASARA Structure software [65]. General view of the dimer of full-length AHK4 in HPt-free form **(top)** and in complex with two AHP2 proteins **(bottom)**, presented in opaque surface view. Hydrogen atoms have been removed. Both models are presented in four projections: "front", "side", "top", i.e., looking at the noncytosolic side of the membrane, and "bottom", i.e., looking at the cytosolic side of the membrane. The "top" and "bottom" views have a scale of 50% relative to the "front" and "side" views. The color codes for domains are shown at the bottom of the illustration. The N-terminal fragments of AHK4 up to the first TM domain have been removed.

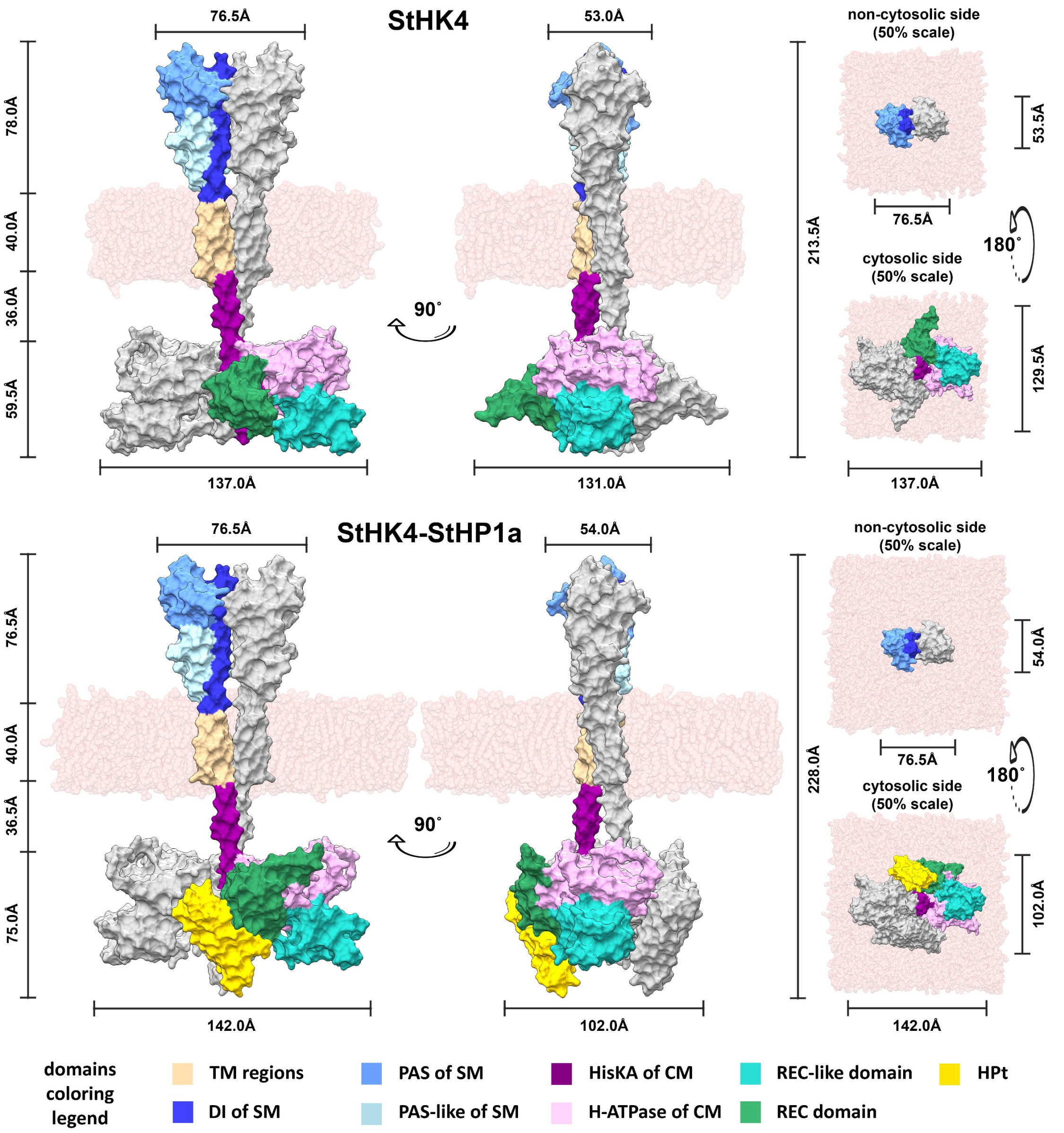

Figure S22. Models of the full-length *Solanum tuberosum* StHK4 receptor obtained using ColabFold [23] and embedded in the membrane in the YASARA Structure software [65]. General view of the dimer of full-length StHK4 in HPt-free form **(top)** and in complex with two StHP1a proteins **(bottom)**, presented in opaque surface view. Hydrogen atoms have been removed. Both models are presented in four projections: "front", "side", "top", i.e., looking at the noncytosolic side of the membrane, and "bottom", i.e., looking at the cytosolic side of the membrane. The "top" and "bottom" views have a scale of 50% relative to the "front" and "side" views. The color codes for domains are shown at the bottom of the illustration. The N-terminal fragments of StHK4 up to the first TM domain have been removed.

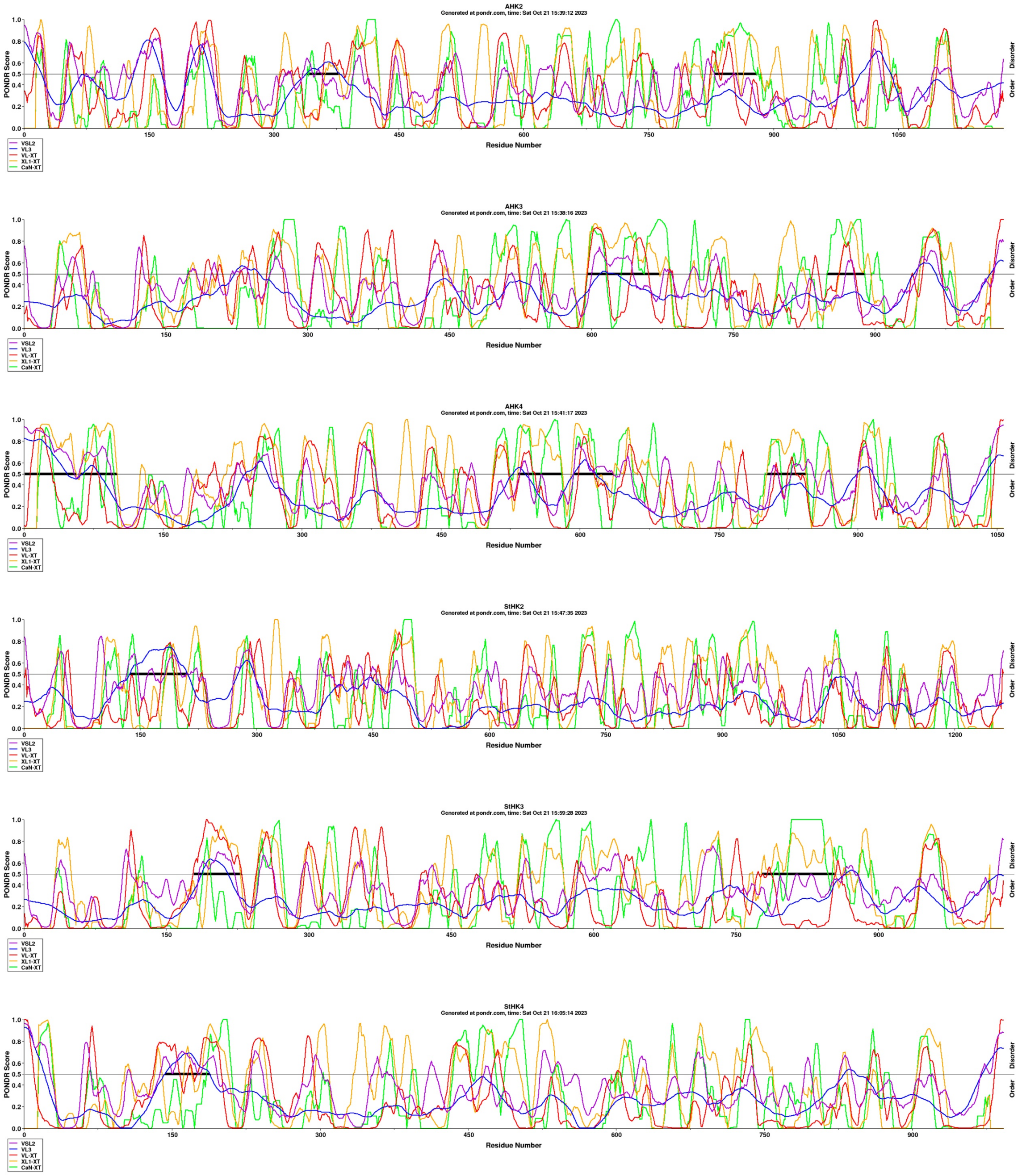

Figure S23. Disorder prediction for AHK2-4 and StHK2-4 receptors performed with PONDR web-server [86]. Graphs displaying the results obtained by different methods (predictors) are colored in different colors: VLXT -red, XL1_XT - yellow, CAN_XT - green, VL3-BA - blue, VSL2 - magenta.

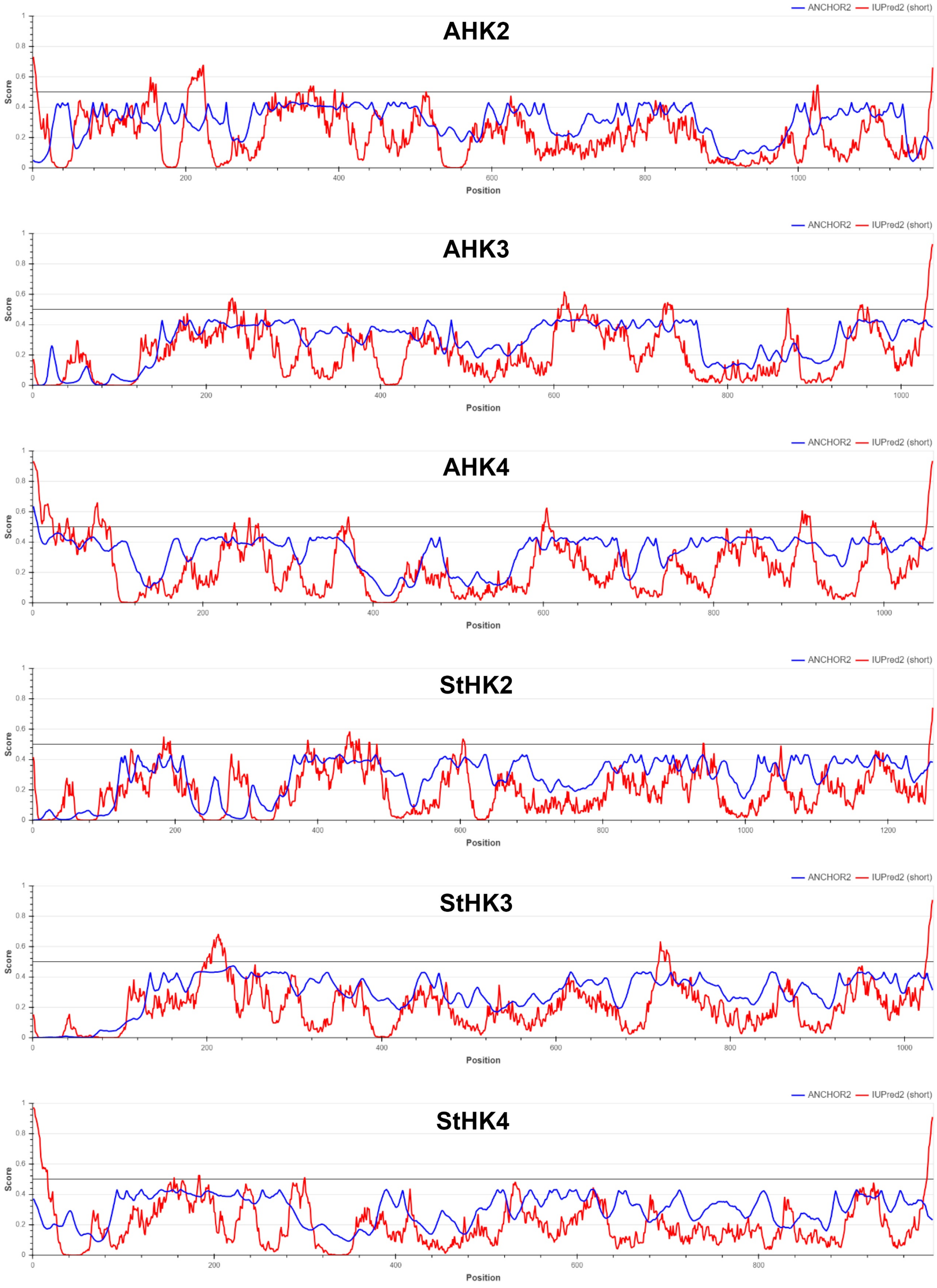

Figure S24. Disorder prediction for AHK2-4 and StHK2-4 receptors performed with IUPred2 web-server [87]. IUPred2 short disorder graphs colored in red, graphs for context-dependent predictions calculated with AHCHOR2 colored in blue.

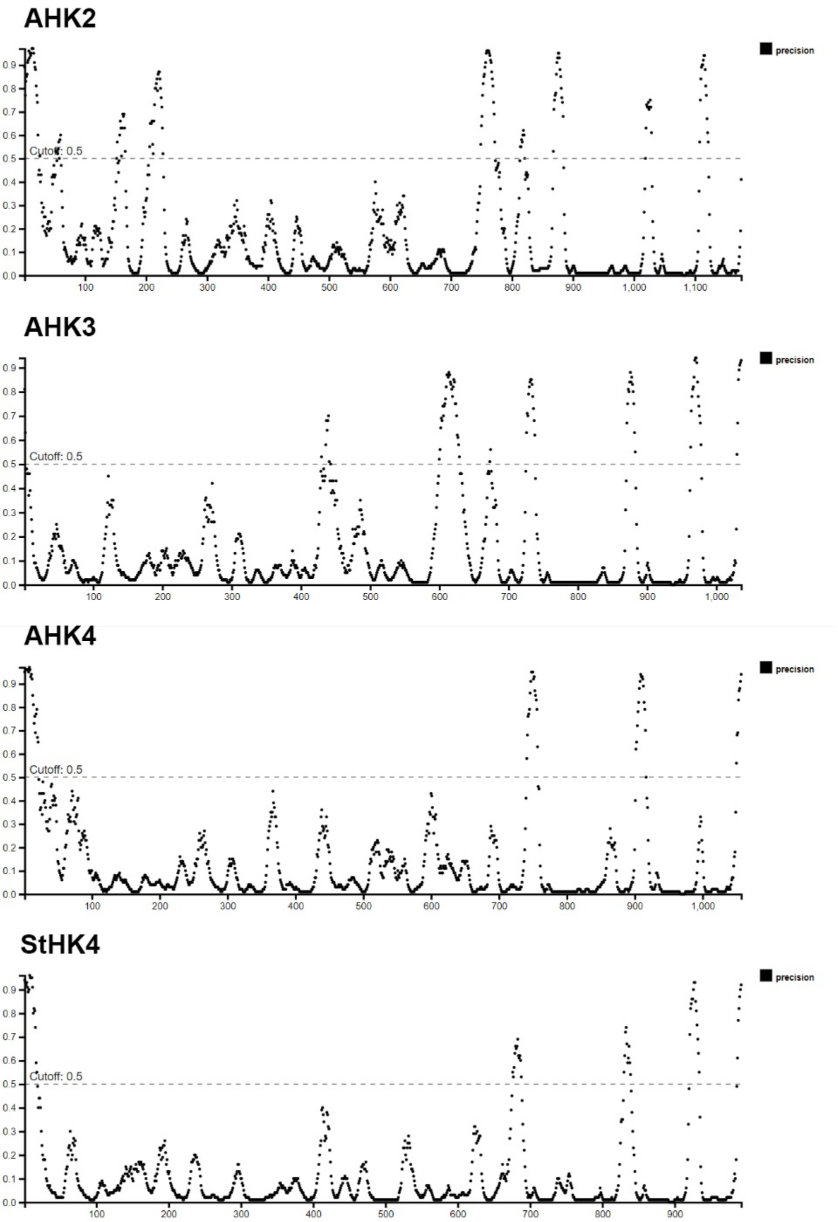

Figure S25. Disorder prediction using DISOPRED 3 software [86] implemented in PSIPRED server [89].

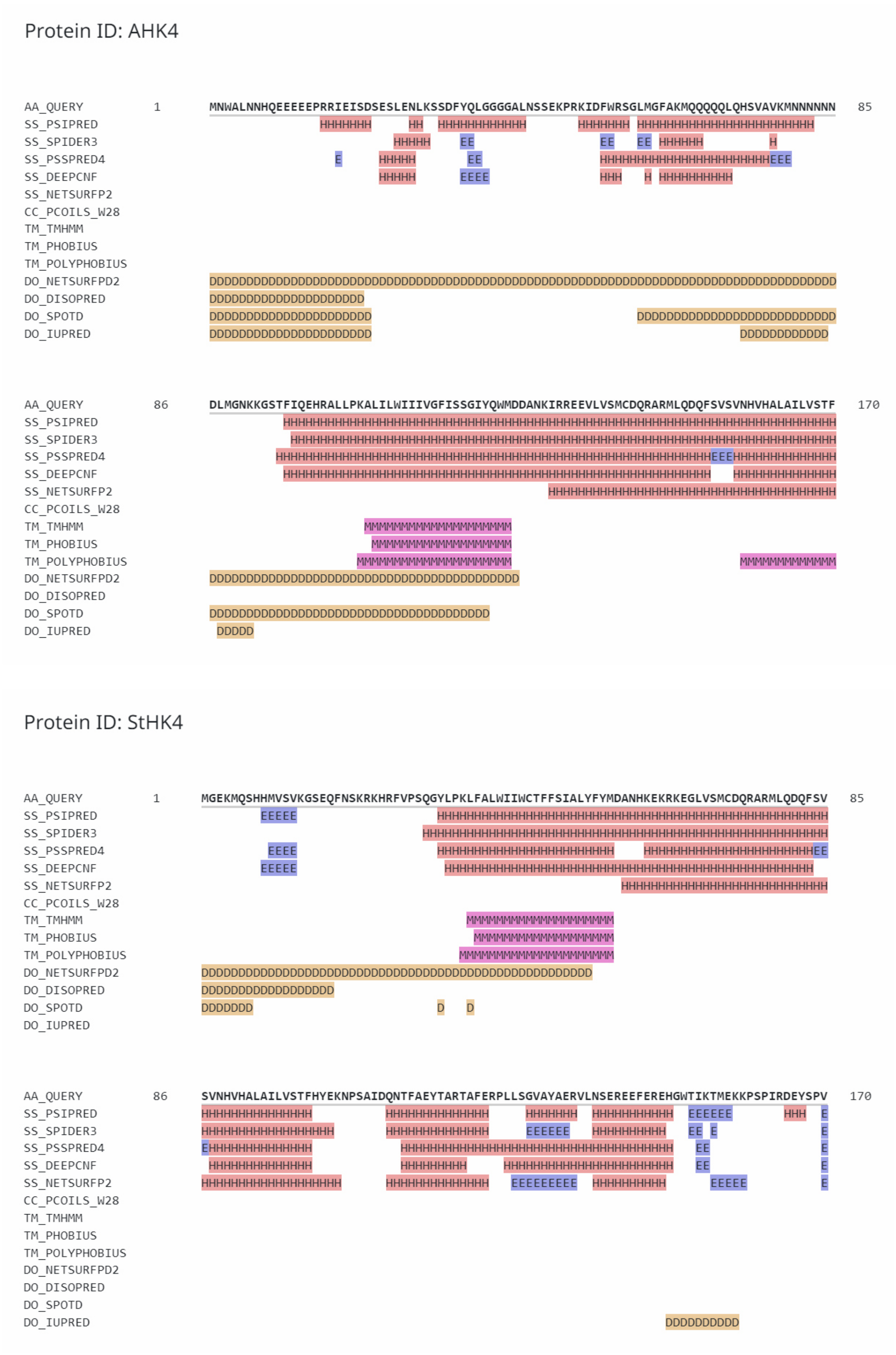

Figure S26. Secondary structure and disorder prediction for AHK4 and StHK4 proteins (N-terminal fragment) with Quick2D web-server [90].

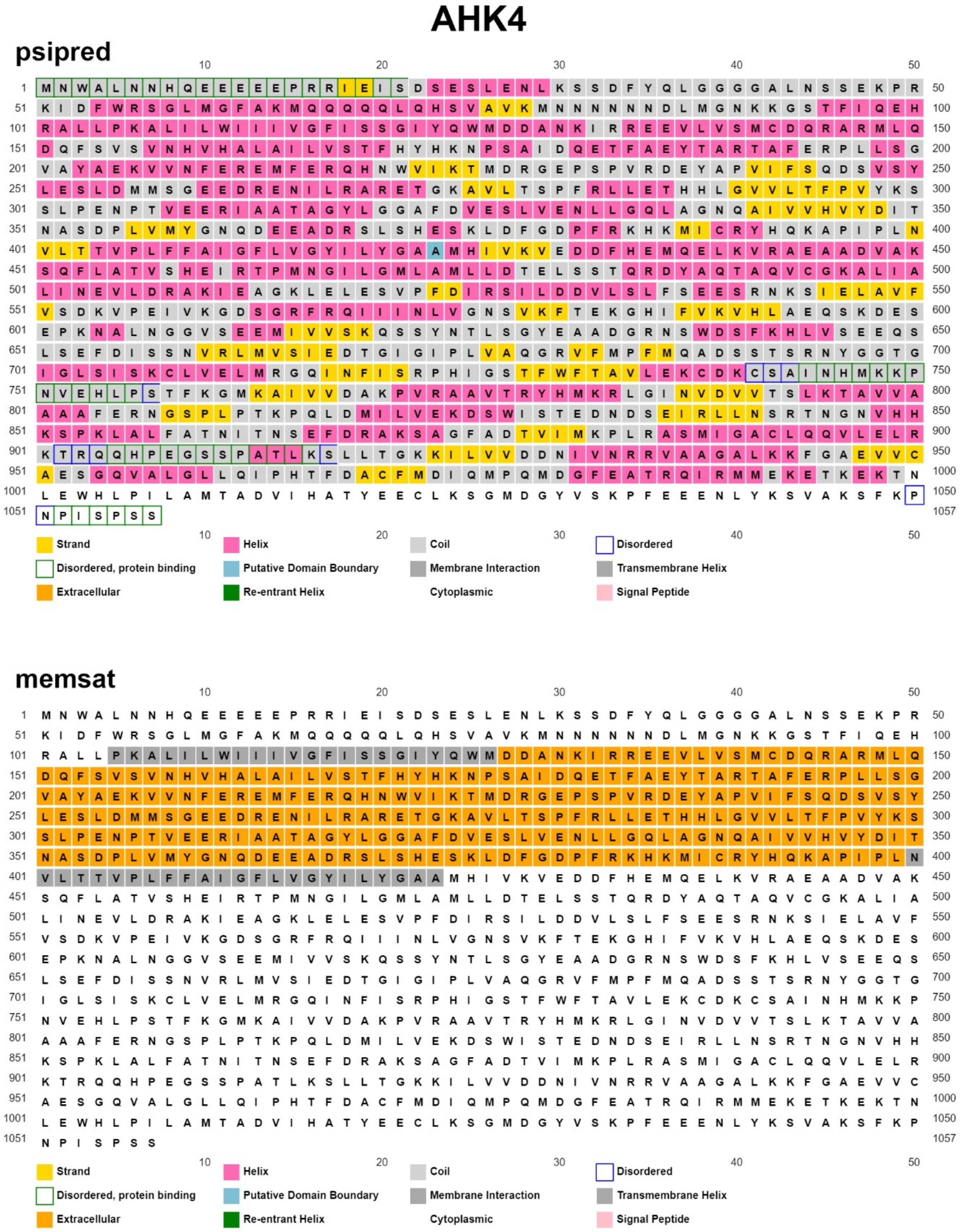

Figure S27. Results of amino acid sequence analysis of the AHK4 receptor with PSIPRED server [89]. Secondary structure prediction (top) was carried out using the PSIPRED method (version 4.0) [91]. Membrane Helix Prediction (bottom) was carried out using MEMSAT-SVM [92]. The color codes for domains are shown at the bottom of the illustration.

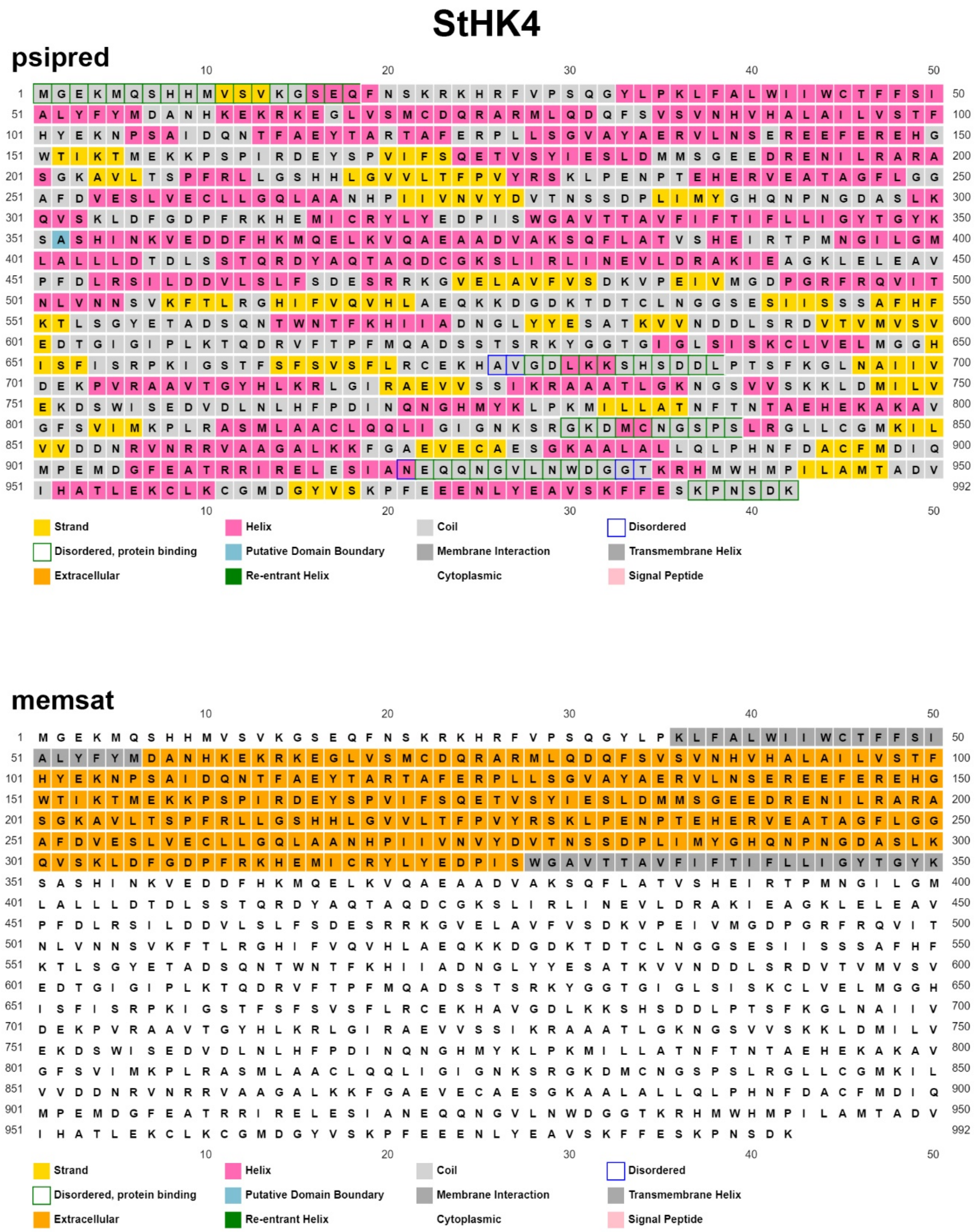

Figure S28. Results of amino acid sequence analysis of the StHK4 receptor with PSIPRED server [89]. Secondary structure prediction (top) was carried out using the PSIPRED method (version 4.0) [91]. Membrane Helix Prediction (bottom) was carried out using MEMSAT-SVM [92]. The color codes for domains are shown at the bottom of the illustration.

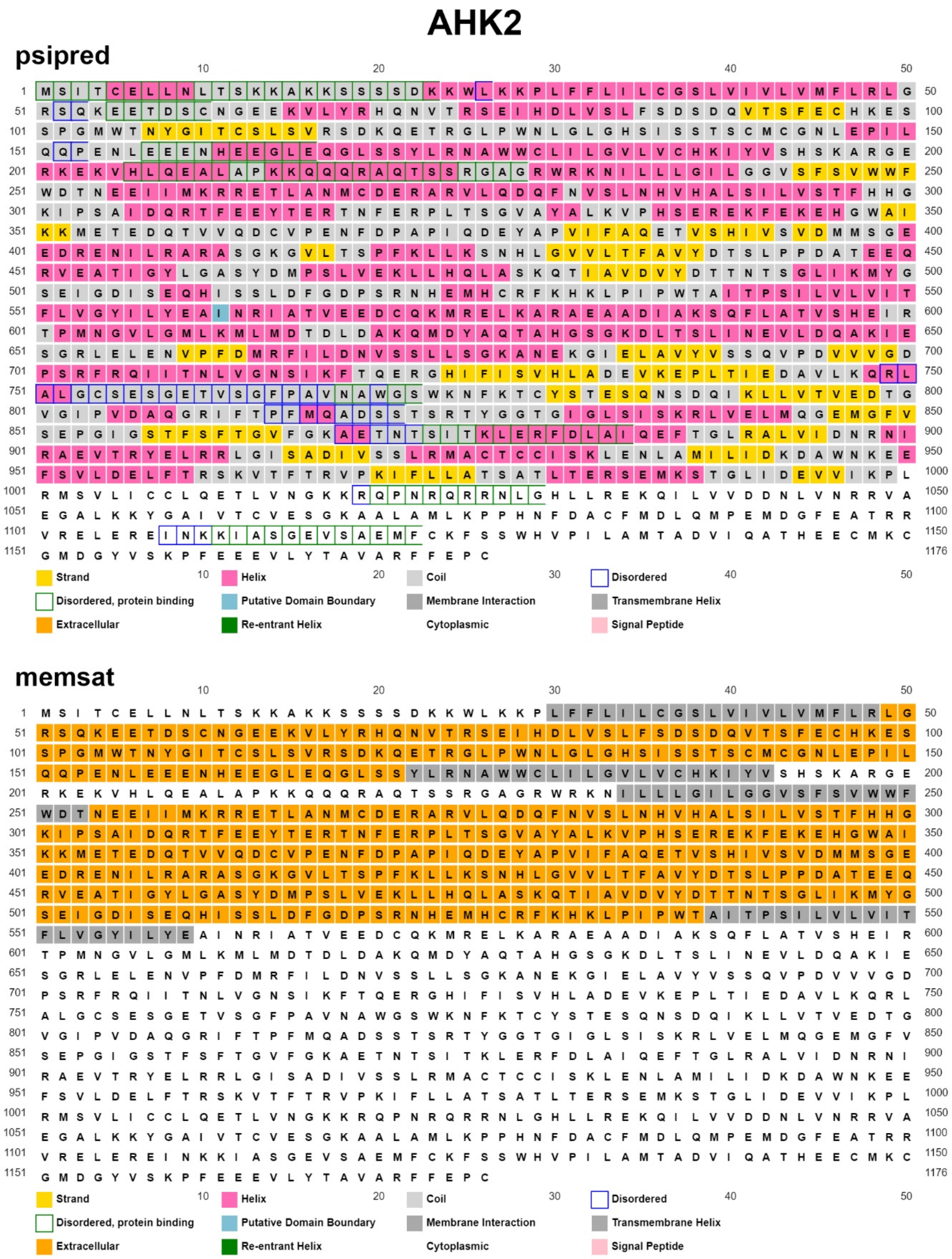

Figure S29. Results of amino acid sequence analysis of the AHK2 receptor with PSIPRED server [89]. Secondary structure prediction (top) was carried out using the PSIPRED method (version 4.0) [91]. Membrane Helix Prediction (bottom) was carried out using MEMSAT-SVM [92]. The color codes for domains are shown at the bottom of the illustration.

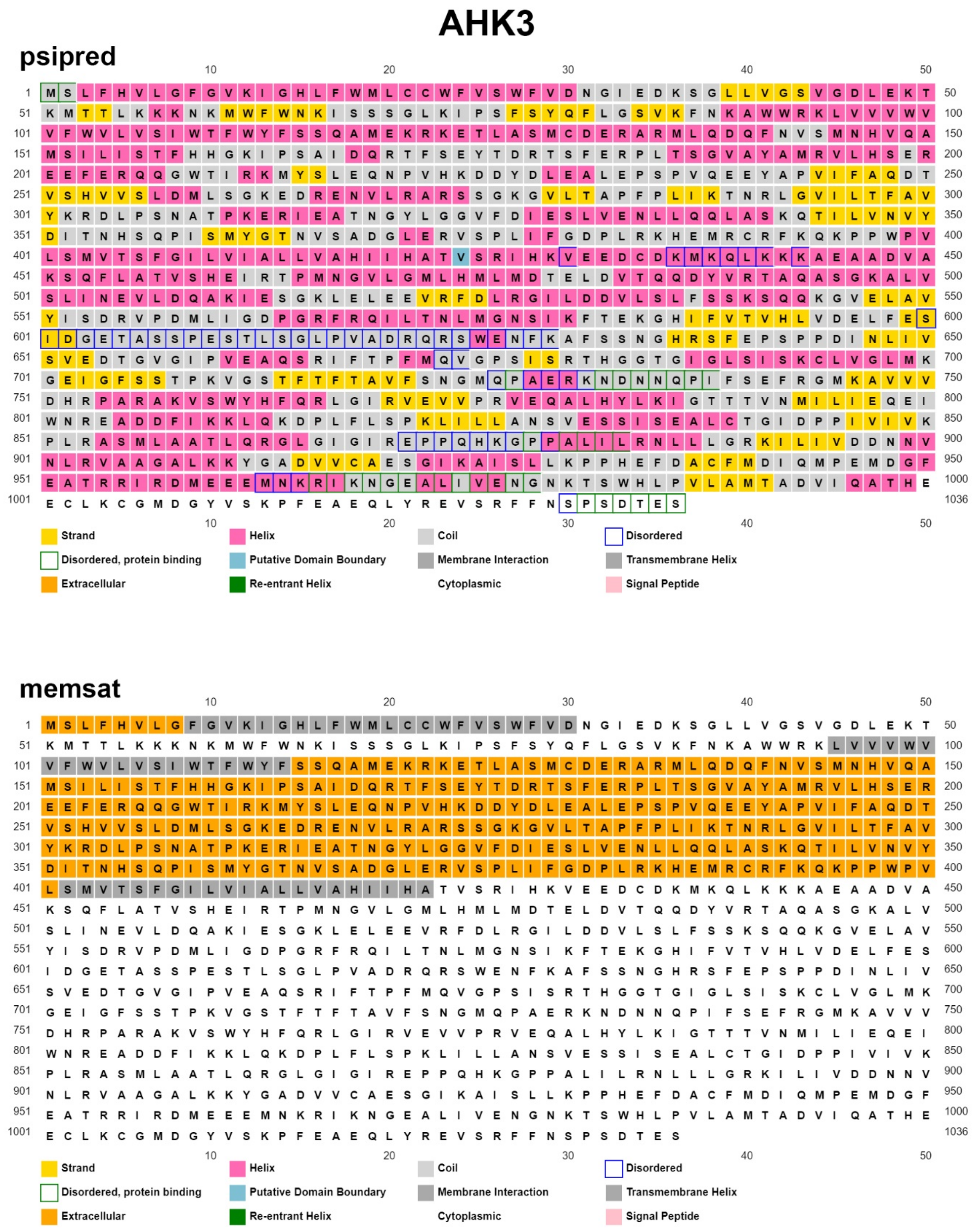

Figure S30. Results of amino acid sequence analysis of the AHK3 receptor with PSIPRED server [89]. Secondary structure prediction (top) was carried out using the PSIPRED method (version 4.0) [91]. Membrane Helix Prediction (bottom) was carried out using MEMSAT-SVM [92]. The color codes for domains are shown at the bottom of the illustration.

Movie S1. Separate file:

Morphing between HPt-free and AHP2-bound conformations of full-sized AHK4 receptor

ColabFold models. Separate files:

Molecular models of full-length *Arabidopsis thaliana* AHK4 and *Solanum tuberosum* StHK4 receptors in different states, obtained in ColabFold and modified as described in the article
